# Comparing substrates for mycelium-based composite insulation materials with thermal and environmental assessment

**DOI:** 10.1038/s41598-026-48045-w

**Published:** 2026-04-14

**Authors:** Joni Wildman, Valeria Cascione, Daniel Henk, Andrew Shea

**Affiliations:** 1 Department of Architecture and Civil Engineering, University of Bath, Bath, UK; 2 Institute of Sustainability and Climate Change, University of Bath, Bath, UK; 3 Milner Centre for Evolution, University of Bath, Bath, UK

**Keywords:** Mycelium-based composite, Bio-based insulation, Biotechnology, Life cycle assessment, Circular economy, Waste valorisation, Engineering, Environmental sciences, Materials science

## Abstract

The construction industry needs to transition toward more sustainable materials to reduce environmental impacts. Insulation materials reduce energy demands of buildings, yet traditional options often have high embodied carbon and rely on finite resources. Mycelium-based composites (MBCs) have emerged as promising bio-based alternatives, formed by the colonisation of fungal mycelium on lignocellulosic feedstocks; the mycelium acts as a natural adhesive and upon drying an inert material is produced. MBCs have demonstrated low thermal conductivity ($\lambda$) and sustainability benefits, with substrate choice being a key factor in determining both thermal and environmental performance. This study emphasises the need for a functional unit (FU) that accounts for thermal performance rather than mass-based declared units (DUs) in Life Cycle Assessment (LCA). MBCs were produced using *Lentinus tigrinus* mycelium and five different substrates: ash-wood chips, bark, beech-wood sawdust, hemp shiv, and wheat straw. Thermal conductivity measurements (ASTM C518) were conducted, revealing that ash-wood chip MBCs exhibited the highest thermal conductivity ($\lambda$ = 0.048 W/m$\cdot$K), while straw-based MBCs had the lowest ($\lambda$ = 0.031 W/m$\cdot$K). Thermal conductivity and density of the material were used to calculate the FU (mass of insulation required to achieve an R-value of 1 m$^2$K/W). A cradle-to-gate LCA (EN 15804) compared environmental impacts per FU for each MBC produced at lab-scale. Ash-wood chip MBCs demonstrated the lowest total global warming potential (GWP) (-9.77 kg CO$_2$ eq), while straw-based MBCs had the highest (4.04 kg CO$_2$ eq). Further analyses examined whether transport distance, waste designation, and carbon sequestration uncertainty would affect substrate ranking and, consequently, selection. While local sourcing and waste-derived substrates reduced emissions, it did not alter rankings. A Monte Carlo analysis confirmed that even with uncertainty in substrate carbon sequestration, MBCs maintained low GWP values. These findings show that low $\lambda$ MBCs can be produced from various substrates. Substrate selection should consider thermal and environmental performance, as carbon sequestration, rather than $\lambda$, is the dominant factor influencing GWP. This underscores the need to consider broader environmental trade-offs when optimising MBC insulation materials.

## Introduction

The construction industry is responsible for approximately 37% of global carbon emissions^1^. There is a critical need for sustainable practices and materials to reduce both operational and embodied carbon emissions in the built environment. Operational carbon refers to the emissions generated during the lifetime of a building, primarily from heating and cooling; this accounts for between 45-80% of a building’s green house gas emissions^2^. Insulation materials play an important role in reducing these energy demands while maintaining comfortable living conditions^3^. Embodied carbon encompasses the emissions associated with the manufacture and disposal of construction materials^2^. While traditional insulation materials are effective at reducing operational carbon, they often have significant embodied carbon footprints, are derived from finite resources, and may pose challenges such as toxicity and flammability^4,5^. Therefore there is a need for alternatives that are both sustainable and effective.

Mycelium-based composites (MBCs) are a class of sustainable materials that have gained interest for insulation applications^6^. These bio-based materials are derived from the cultivation of fungal mycelium on lignocellulosic substrates with mycelium being the vegetative structures in fungi consisting of a network of filamentous cells called hyphae^7,8^. During growth, the mycelium colonises and digests the substrate and in doing so acts as a natural adhesive. The colonised substrate is then dried to kill the fungus, resulting in an inert bio-based material^9^. MBCs have attracted attention for their favourable environmental properties, including low embodied carbon, biodegradability, non-toxicity, and the ability to valorise various waste streams such as agricultural waste^7,10^. Additionally, these materials exhibit low thermal conductivities, which make them of interest for use in insulation applications^11^.

Thermal conductivity ($\lambda$) is a key parameter for evaluating insulation materials and can be defined by Fourier’s law of conduction^12,13^. Lower $\lambda$ values indicate better insulating performance, with values below 0.1 W/m$\cdot$K typically considered good insulation materials^14^.

Materials with lower thermal conductivity require reduced thickness to achieve the same thermal resistance (R-value), offering both practical and economic advantages. Conventional insulation materials such as expanded polystyrene (EPS), extruded polystyrene (XPS), and mineral wool have thermal conductivities of approximately 0.032 W/m$\cdot$K, 0.03 W/m$\cdot$K, and 0.04 W/m$\cdot$K, respectively^15–17^. MBCs have demonstrated comparable thermal conductivities (ranging between 0.026 W/m$\cdot$K to 0.18 W/m$\cdot$K), though their performance can vary depending on factors such as substrate type, fungal species, and manufacturing processes^11^.

Important properties of MBCs that require optimising include their thermal properties to make their thermal conductivity more competitive against traditional materials^18^. Optimising the thermal properties of the MBC will also improve environmental impacts by reducing associated operational impacts and reducing the amount of MBC required for a given reduction in heat transfer. One factor that affects thermal properties and environmental impacts is the choice of substrate^10^. Previous research has highlighted variability in $\lambda$ and environmental impacts based on the substrate used, yet comprehensive comparative studies remain limited^10^.

MBCs have been developed using a variety of lignocellulosic substrates^19^. This demonstrates their adaptability to diverse waste streams and regional resources. Commonly used substrates include agricultural by-products such as wheat straw, hemp shiv, rice husks, and flax, as well as processed materials like wood chips and cellulose. Examples of values obtained for different substrates are straw based substrates have yielded thermal conductivities in the range 0.026-0.081 W/m$\cdot$K^20–24^, wood-chip based MBCs in the range 0.032-0.043 W/m$\cdot$K^25,26^, and sawdust based MBCs in the range 0.04-0.07 W/m$\cdot$K^26–29^. These ranges provide examples of figures obtained for different substrates, though meaningful comparisons on thermal performance are best made between studies using similar measurement methodologies due to different uncertainties associated with different measurement techniques^11^. Elsacker et al. measured $\lambda$ of composites based on fibres from flax, hemp and straw and obtained thermal conductivities of 0.0578 W/m$\cdot$K, 0.0404 W/m$\cdot$K, and 0.0419 W/m$\cdot$K respectively, made using *Trametes versicolor*^20^. Tsao et al. also studied the effect of using different substrates on thermal conductivity, testing composites made with varying ratios of rapeseed straw and cellulose^30^. They found that the pure rapeseed straw composites exhibited the lowest thermal conductivity of 0.057 W/m$\cdot$K, while the pure cellulose composite had higher thermal conductivity of 0.085 W/m$\cdot$K. These results demonstrate how the composition of substrates influences the thermal performance of MBCs. Wildman et al. reviewed the effect of substrate, as well as other variables such as fungal species, density, and moisture content, on measured thermal conductivity of MBCs. These reviewed results demonstrate that substrate choice is an important variable in determining measured thermal conductivity^11^.

In addition to optimising thermal performance of MBCs, the environmental impacts need to be carefully assessed. This can be done using life cycle assessments (LCA), a method for quantifying the environmental effects of a product across different stages of their life^31^.

A number of LCA studies have been conducted on different MBCs, focusing on different aspects of their development and using different functional units (FU) or declared unit (DU) in the assessment. The functional unit (FU) is a quantified measure of the performance of a product or process, providing a basis for comparing environmental impacts relative to a specific function. In contrast, a declared unit is used when a functional comparison is not defined, where impacts are reported per unit of material or product (e.g. per unit mass)^32^. Table 1 shows the studies conducted to date, outlining the use case, material properties (where relevant), as well as the functional unit or declared unit used. Whilst a functional unit quantifies the performance of a product to enable equivalent comparisons in LCA studies, declared units are sometimes used when a functional basis is not defined^33^. Here, A1-A3 refers to cradle-to-gate, and A-D to full life-cycle assessment stages (cradle-to-grave) as defined in EN 15804^32^.

**Table 1:** Table showing the LCA studies on mycelium-based composites and mycelium materials. Detailed are the use application for each material, the functional unit (FU) or declared unit (DU) chosen, the relevant material properties ($\lambda$ and $\rho$), the standard used, and the scope of the LCA study. A1-A3 refers to cradle-to-gate, and A-D to full life-cycle assessment stages (cradle-to-grave) as defined in EN 15804.

Material description	FU/ DU	Lambda	Density	Standard	Scope	References
MBC insulation	10 cm $\times$ 10 cm $\times$ 10 cm block	0.05	212	EN15804	A1-A3	^34^
	1 m$^2$ of material, depth to give thermal resistance value of 1 m$^2$ K/W and a lifetime of 30 years, equivalent to 11 kg of MBC material	0.05	212	EN15804	A1-D	^34^
	1m$^3$ material	0.04	163		A1-A3	^35^
	1 kg of MBC			EN15804	A1-A3	^36^
	24 cm $\times$ 11.5 cm $\times$ 7.1 cm MBC			ISO14040/44		^37^
	R-value of 6.3 m$^2$ K/W with thickness 510 mm				A1-A3	^38^
	1 m$^2$ with thickness to give a U-value 0.05 W/m$^\text {2}$K	0.05	229	EN15804	A1-A3	^39^
	(10 $\times$ 10 $\times$ 10 cm), equivalent to 0.212 kg of mycelium-derived material (intended for an area of 1 m$^2$ designed to provide an R-value of 4 m$^2$K/W and a lifespan of 30 years)					^6^
	R value between 0.15 and 0.20 W/m$^\text {2}$K, and 1 m$^2$ area	0.08	99	EN15804	A1-D	^40^
MBC block	1 m$^3$ material		300	ISO14040/44	A-D	^41^
	29.5 cm $\times$ 14 cm $\times$ 9 cm mycelial brick			ISO14040/44	A-D	^10^
	10 cm $\times$ 10 cm $\times$ 10 cm MBC block				A-D	^42^
MBC acoustic panel	1 m$^2$ of mycelium			ISO14040/44	A-D	^43^
MBC packaging insert	Service shipping box with dimensions of 31.12 cm $\times$ 30.48 cm $\times$ 15.24 cm, 1.4 kg			ISO14040/44	A-D	^44^
MBC leather	1 m$^2$ mycelium leather			ISO14040/44	A-D	^45^
	1 m$^2$ mycelium leather			ISO14040/44	A1-A3	^46^

These studies have provided several insights into the environmental impacts of MBCs. Alaux et al. demonstrated that scaling up production significantly reduces environmental impacts due to improved energy efficiencies^34^. This is important to consider when comparing with established insulation materials, such as EPS and XPS, as many MBCs are still produced in lab-scale environments for research purposes. Alaux et al. assessed a 10 cm $\times$ 10 cm $\times$ 10 cm block of MBC insulation with a thermal resistance of 1 m$^2$K/W and a lifespan of 30 years, furthermore highlighted the importance of energy optimisation during the manufacturing process^34^. Similarly, Livne et al. found that incubation time was a key driver of embodied energy (EE) and embodied carbon (EC) in their LCA of 1 m$^3$ of MBC material due to the energy use to maintain a moderate incubation temperature^35^. The inclusion of carbon exchange measurements in their study added further insight into the biogenic carbon dynamics involved in MBC production, and they concluded that their MBCs could act as carbon sinks during their lifetime. These LCAs, among others, emphasized the importance of minimising energy consumption during incubation and drying processes, where possible, and prioritising the use of renewable energy sources. However, transitioning to more sustainable energy options is not always practical, as energy grids are often fixed for a given lab or factory^47^. Therefore, it is crucial to explore alternative strategies within researchers’ control to reduce both embodied and operational energy^48^.

Comparative studies that integrate scenarios to reflect variability in production and usage provide further insights into the environmental impacts of MBCs. Cascione et al. conducted an LCA comparing various bio-based insulation materials, including MBCs, under best, worst, and baseline scenarios^40^. Their findings highlighted the environmental benefits of MBCs in ideal conditions while emphasising the trade-offs associated with less favourable scenarios, such as energy-intensive production methods or suboptimal end-of-life management.

The treatment of biogenic carbon throughout the life cycle of a material can add further complexity, which is necessary when assessing MBCs^49^. Maierhofer et al. examined the differences between static and dynamic carbon accounting, highlighting how temporal dynamics can significantly alter the perceived environmental benefits of biogenic materials^42^. These findings are particularly relevant in contexts where the release and sequestration of biogenic carbon are influenced by production processes and substrate decomposition rates.

Considering the implications of substrate choice, Volk et al. compared the impacts of using hemp and sawdust substrates, revealing significant variations in environmental outcomes^36^. However, their reliance on a mass-based declared unit did not account for differences in $\lambda$ or density ($\rho$) and therefore limits the applicability of the findings in the context of insulation applications. Also comparing substrates, Stelzer et al. employed a performance-based FU targeting an R-value of 4 over a 30-year lifespan but assumed the substrates were interchangeable in terms of performance, failing to account for differences in substrate-specific properties (e.g. $\lambda$ and $\rho$)^37^.

Although these studies have revealed several insights about environmental aspects of MBCs, direct comparasions of LCA results from different MBCs are complicated by differences in the selection of functional units (FUs) or the use of declared unit, the latter often using mass-based metrics without accounting for material performance. This makes direct comparisons between MBCs and conventional insulation materials limited, and selecting substrates by comparing these studies also difficult. The use of mass as a FU does however have some advantages, as MBCs have alternative applications to insulation materials (e.g. packaging) and therefore adopting a broader FU can be better in a development stage where end use is uncertain. Nevertheless, in future assessments focused on insulation applications, FUs that reflect thermal performance would be instructive.

In summary, substrate selection plays an important role in determining the environmental and functional performance of MBCs, making it a crucial consideration for researchers and material developers. However, further quantitative analyses and detailed LCAs are necessary to fully understand this impact, particularly to integrate the effects of thermal performance and environmental factors for more targeted optimisation efforts. The thermal performance of different materials is accounted for with the appropriate selection of functional unit within the LCA framework, whereas adopting a declared unit based on mass or volume does not account for differences in thermal performance. It is also important to evaluate the environmental trade-offs involved in enhancing $\lambda$ (e.g., through substrate modification or alternative processing methods) against the associated environmental costs. Different substrates may be more suitable in certain situations, such as when they are locally available and require minimal transportation. This aspect has yet to be quantified to guide substrate selection. This research seeks to address these unresolved issues by examining how substrate choice affects both the functional and environmental performance of mycelium-based composites.

The aims and objectives of this study are as follows:

- To produce and thermally characterise mycelium composites using five different substrates.
- To conduct a life cycle assessment (LCA) to evaluate the environmental impacts of MBCs produced using different substrates.
- To explore how variations in thermal performance impact the functional unit and LCA results, helping to select the optimal substrate from the five options.
- To demonstrate the role of balancing thermal performance with environmental considerations in choosing the most suitable substrate for mycelium composites.
- To assess the extent to which different factors affect the choice of the most suitable substrate based on global warming potential. The factors considered here are the regional availability of substrates, the waste designation of the substrate (waste vs. non waste), and the carbon sequestration ability of the substrate.

## Materials and method

### Mycelium-based composite production

#### Substrate preparation

The following substrates were used to make MBCs: Ash-wood chips, bark, hemp shiv, wheat straw, and beech-wood sawdust. The ash-wood chip was of dimensions approximately 10 $\times$ 10 $\times$ 5 mm with a wood density of 610 kg/m$^3$. The bark was fibrous in nature and density 240 kg/m$^3$. The hemp shiv was of dimensions 10 $\times$ 5 $\times$ 2 mm and density 110 kg/m$^3$. The straw was of length approximately 30 mm and diameter 3 mm. The beech-wood sawdust was in particles of dimensions approximately 1 x 0.5 x 0.5 mm and density 680 kg/m$^3$. All substrates were obtained in the dimensions described without additional processing required to produce the MBC specimens.

The processed substrates were placed in individual buckets and soaked in water for 24 h. The substrates were weighed before and after soaking to determine water absorption. This step was necessary to ensure that the substrates reached adequate moisture content to support fungal growth. Subsequently, the substrates were drained and mixed with plain flour (1:10 ratio flour to substrate) to serve as an additional nutrition source for more rapid fungal growth. This mix was packed into an autoclavable bag before being sterilised in an autoclave.

#### Fungal culture preparation

A liquid culture inoculum of *Lentinus tigrinus* was obtained from kingdomLCs. The identity of the species was verified based on sequencing of the ITS4 region. The inoculum was subsequently propagated, maintained, and stored on malt extract agar with yeast extract (MEYA). Mycelium growing on MEYA was then used to inoculate hydrated and sterilised brown rice. The fungi was allowed to colonise the rice for 5 days in an incubator at 23 $^{\circ }$C. The grain colonised by mycelium was subsequently used as grain spawn to form the composites.

#### Composite production

In a biosafety cabinet the substrate and grain spawn were combined in a ratio of 10:1 with the grain spawn broken up into mycelium-colonised individual grains. A large bowl and spoon, cleaned with 70% isopropanol, were used to mix the spawn and substrate.

The mixture was then packed in to cylindrical moulds (height 50 mm, diameter 80 mm) cleaned with 70% isopropanol. Once the moulds were filled they were covered with a sheet of parafilm secured with tape to allow for air exchange whilst minimising contamination during incubation.

The specimens were then incubated at 23 $^{\circ }$C for 5 days. The specimens were then removed from the moulds and weighed. They were then dried in an oven at 50 $^{\circ }$C for 48 h. The specimens were weighed after 40 h and after 48 h to ensure that no further mass was lost upon 8 h additional drying thus the specimens were not retaining moisture. Four biological repeats were made for each substrate to give total of 20 specimens.

### Thermal characterisation

The thermal conductivity of each specimen was determined using a Heat Flow Meter according to ASTM-C518^50^. Five thermal analysis tests were performed on each of the 20 specimen (100 tests in total) using a Thermtest HFM-25. Each specimen was stored in the laboratory at room conditions for 1 week (approx. 20 $^{\circ }$C and 50% RH) prior to testing. Specimen density was also calculated according to ASTM C303 prior to testing^51^. The test was conducted at a mean temperature of 20 $^{\circ }$C with a 20 $^{\circ }$C temperature difference between the plates.

### LCA

#### Goal and scope

The LCA was completed in alignment with EN 15804:2012+A2:2019^32^. The goal of this study is to evaluate the environmental impacts of mycelium-based composites used for insulation applications, produced using five different substrates. The analysis will consider thermal performance and material properties, such as density, to calculate the functional unit (in kilograms); the substrate-specific environmental factors per kilogram (e.g., carbon sequestration and material-related impacts) can then be included in the assessment. This will inform substrate selection strategies for optimised environmental outcomes, considering both operational and embodied carbon. Additionally, the study considers various transport scenarios to examine how regional availability and proximity to substrate sources influence environmentally driven decision-making in substrate selection.

**Fig. 1:**
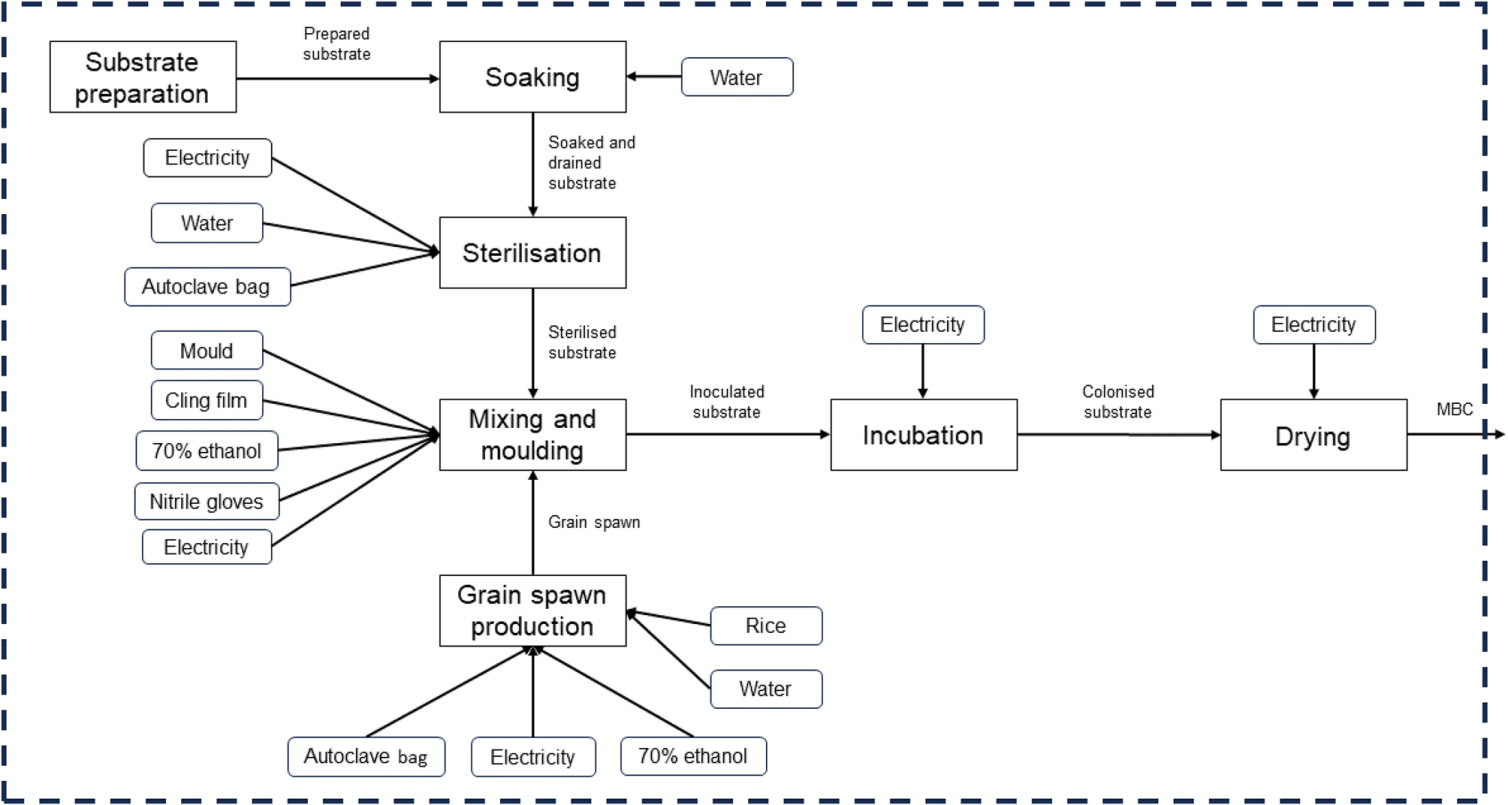
Figure showing the system boundaries adopted in the LCA of mycelium-based composites produced using different substrate materials and *Lentinus tigrinus* grain spawn. The boundaries include the substrate preparation and the mycelium-based composite production (A1-A3 stages). The different stages of the mycelium-based composite production process as shown in square boxes, with the inputs at each stage shown in rounded boxes. The substrate preparation inputs for each substrate considered in this study (ash-wood chips, bark, beech sawdust, hemp shiv, and wheat straw) are shown in Fig. 2.

**Fig. 2:**
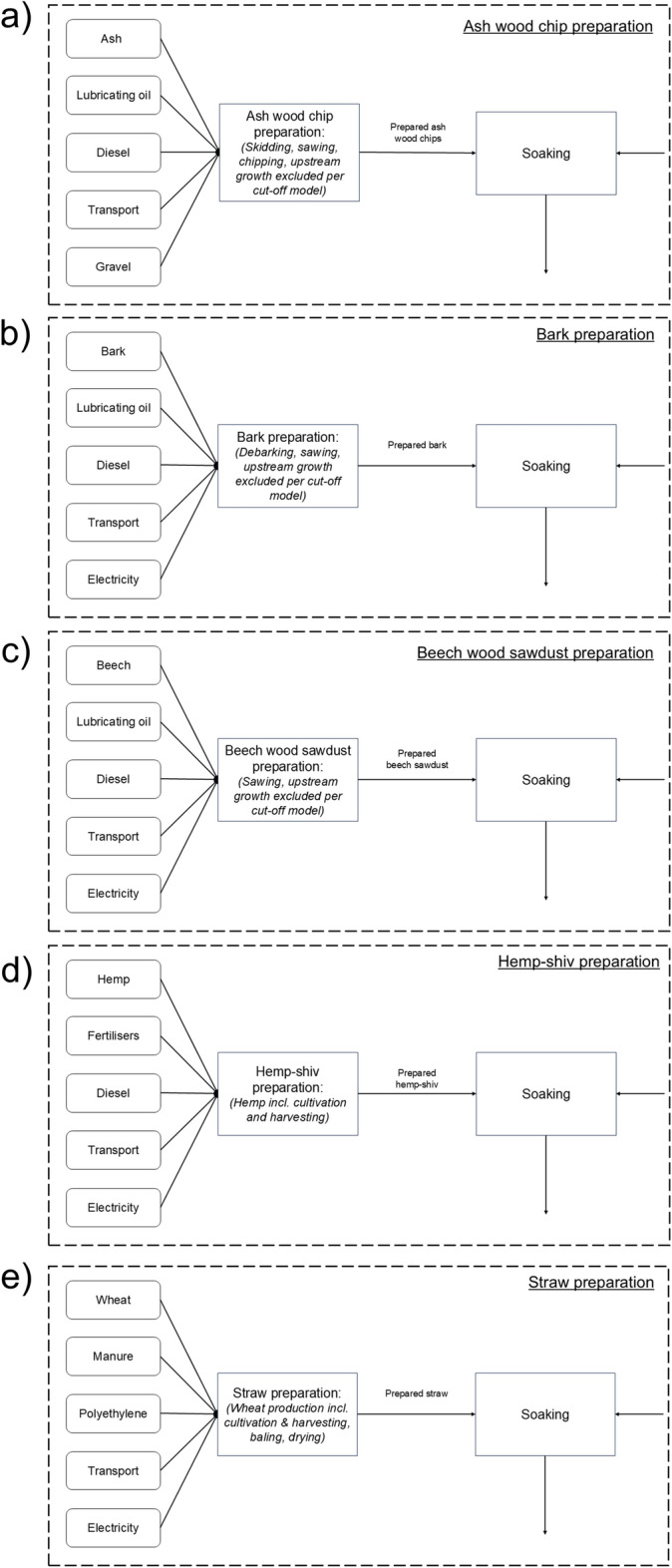
Figure showing the inputs associated with the substrate preparation stage (feeding into the system depicted in Fig. 1) for each substrate considered in this study (ash-wood chips, bark, beech sawdust, hemp shiv, and wheat straw). Agricultural feedstocks (hemp shiv and wheat straw) include cultivation and harvesting processes, while forestry-derived materials (ash wood chips, beech wood chips, and bark chips) are modelled as residues under the Ecoinvent v3.6 cut-off system model, with upstream forest growth burdens allocated to primary wood products.

The system boundaries and processes considered for evaluating the production of mycelium-based composites are depicted in fig. 1, with the substrate specific processes illustrated in fig. 2. The boundaries encompass the substrate preparation stage, which varies between the five different substrates. Agricultural feedstocks (hemp shiv and wheat straw) include cultivation and harvesting processes, while forestry-derived materials (ash wood chips, beech wood chips, and bark chips) are modelled as residues under the Ecoinvent v3.6 cut-off system model, with upstream forest growth burdens allocated to primary wood products. Once prepared, the stages are common to all substrate scenarios, representing the mycelium-based composite production process. Although the stages remain consistent, the inventory values for the inputs differ depending on the substrate used. These processes include substrate soaking, substrate sterilisation, grain spawn production, mixing of substrate and grain spawn, incubation, and drying. Collectively, these steps correspond to the stages A1-A3 of the life cycle (from cradle to gate). This study focuses on the cradle-to-gate impacts of MBCs due to the limited availability of reliable data for the use and end-of-life phases. Additionally, the primary aim is to examine how variations in thermal performance influence substrate selection when defining the functional unit, while also assessing the effects of transport scenarios and carbon sequestration, to inform material optimisation efforts.

The functional unit (FU) of this LCA study is defined as the mass (kg) of MBC insulation required to achieve a thermal resistance (R-value) of 1 m$^2$K/W over an area of 1 m$^2$. The relationship between these is expressed by the equation:

$$FU = R\lambda \rho A$$

where: R is the thermal resistance (equal to 1 (m$^2$K/W)), $\lambda$ is the thermal conductivity in W/(m$\cdot$K), $\rho$ is the density in kg/m$^3$, and A is the area (equal to 1 m$^2$)^52^. This FU choice quantifies the volume of insulation material needed to achieve the specified thermal resistance. Table 2 presents the mass calculations of the functional unit across the five substrate scenarios, based on measured values of $\lambda$ and $\rho$. It is assumed that $\lambda$ and $\rho$ are the same for composites made with A equal to 1 m$^2$ as for those produced in this study.

#### Inventory

Details of the life cycle inventory (LCI) can be found tabulated in the n Information. The LCI was primarily based on laboratory measurements. Where direct measurements were not possible, the data were supplemented using literature sources and technical data sheets^53–56^. Background data were sourced from the Ecoinvent V3.6 database^57^. OpenLCA^58^ was used for the LCA analysis. Background datasets for agricultural and forestry feedstocks (e.g., hemp, wheat straw, wood residues) were sourced directly from Ecoinvent v3.6 using the cut-off system model. Under this attributional modelling framework, allocation of upstream burdens between primary products and co-products is handled within the database. In the cut-off model, residues entering the technosphere (e.g., straw, bark, sawdust) are considered burden-free with respect to upstream cultivation and harvesting impacts, and only subsequent processing and transport are included. No additional allocation procedures were applied in this study beyond those inherent to the selected system model. In the waste scenario, feedstocks were modelled as burden-free at the point of entry to the system, consistent with cut-off modelling assumptions.

Biogenic carbon was considered by applying the static -1/+1 approach, following the current version of the EN 15804 standard (CEN 2019)^32^. The quantification of the biogenic carbon contained in organic materials, including ash-wood chips, bark, beech-wood sawdust, hemp, and wheat straw, is based on the values in the literature for their respective carbon content^59–61^.

To calculate the amount of CO$_2$ sequestered in these organic substrates, the molar masses of carbon (C) and carbon dioxide (CO$_2$) are considered. CO$_2$ has a molar mass of 44 g per mole, while C has a molar mass of 12 g per mole, resulting in a mass ratio of CO$_2$ to C of 44/12 = 3.67^62^. Using this ratio, and the substrate-specific carbon content, the mass of CO$_2$ assumed to be sequestered per kg of each substrate was: 1.76 kg CO$_2$/kg for ash-wood chip, 1.83 kg CO$_2$/kg for bark, 1.72 kg CO$_2$/kg for beech-wood sawdust, 1.5 kg CO$_2$/kg for hemp, and 1.29 kg CO$_2$/kg for wheat straw.

These values were then integrated into the life cycle inventory as carbon inputs to represent the biogenic carbon sequestered in the materials following the EN16449 standard^62^. The percentage carbon contents used in this study were sourced from the literature^59–61^. However, these values are subject to uncertainty due to variations in the biological material, including species differences and growing conditions^59^. To assess the influence of this uncertainty on the overall environmental impact of the materials, a Monte Carlo simulation was performed using the OpenLCA software for each of the five MBCs. Monte Carlo simulation is a statistical technique that uses repeated random sampling to assess the variability of an outcome based on uncertain input parameters. Numerous iterations are run with the input sampled such that the variability in the outcome can be estimated^63^. In this analysis, the carbon dioxide input in the simulation (calculated based on the substrate carbon sequestration) was modelled as a normally distributed variable with a standard deviation of 1. A total of 10000 simulation runs were conducted for each MBC. The results were then analysed by calculating the mean impact, standard deviation, 5th and 95th percentiles, and median to quantify the variability in outcomes.

#### Transport scenarios

To evaluate how the local availability of substrates determines optimal substrate selection, four transport scenarios were considered. The base case assumed the substrate was locally sourced (10 km transport by lorry). The transport scenarios considered were as follows:

1. Transport over 10 km by lorry
2. Transport over 100 km by lorry
3. Transport over 150 km by lorry and 200 km by rail
4. Transport over 150 km by lorry and 300 km by ship

These scenarios were chosen to cover a range of realistic supply chain configurations for sourcing substrates for MBC production. For the lorry and rail scenario, the substrate was assumed to originate from France, while for the lorry and ship scenario, the source location was set as the Netherlands.

#### Waste scenarios

The substrates used in this study, specifically agricultural waste, can be classified as either waste or non-waste, which influences their treatment within the LCA framework. When classified as waste, it is assumed that the material has already been discarded and that its previous life-cycle burdens are not carried over. In the LCA approach previously described, the substrates were not treated as pure waste, meaning that the impacts associated with their processing were included. To assess the influence of waste classification on the environmental impacts, the LCA was repeated with the substrates designated as waste. Under this scenario, only transport emissions and carbon sequestration were included in the input calculations, while upstream processing impacts were excluded. This designation primarily affects the substrate preparation stage, while the subsequent MBC production stages remain unchanged.

## Results and discussion

### Composites

**Fig. 3:**
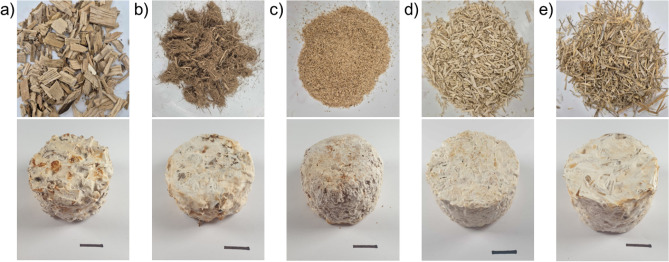
Figure showing the five different raw substrates used to produce the mycelium-based composites (MBC) in this study (top row) and an example of a MBC specimen produced using each of the five different substrates (bottom row). The substrates and specimens are: (**a**) ash-wood chips with an ash-wood chip based MBC, (**b**) bark with a bark based MBC, (**c**) beech-wood sawdust with a beech-wood sawdust MBC, (**d**) hemp shivs with a hemp shiv based MBC, and (**e**) wheat straw with a wheat straw based MBC. For each substrate four repeats were produced to give 20 specimens in total.

Figure 3 shows the MBCs produced using *Lentinus tigrinus* for this study. Four repeat composites were successfully produced using each of the five substrates, resulting in a total of 20 specimens. Across the different samples, the fungus demonstrated good colonisation and homogeneous growth. All composites displayed good structural integrity and were easily handled. Variation between the substrates included difference in colouration, with the ash-wood chip MBCs having reddish patches, as well as differences in the development of a fungal skin layer with the straw and bark substrates giving rise to a more distinct skin layer compared to the other substrates.

When comparing the growth rates of *Lentinus tigrinus*, all substrates exhibited the same colonisation time of five days during incubation. While this uniformity was observed for the substrates tested in this study, it may not hold true for all substrate-fungus combinations. Variations in incubation time, driven by differences in colonisation rate and efficiency, could lead to additional environmental impacts, particularly due to the energy demands of prolonged incubation periods.

The presence or absence of a fungal skin is another notable observation. Bark and straw substrates yielded composites with the most prominent fungal skins. While these skins were relatively small in this study, they could influence properties such as the thermal conductivity of the bulk material. In certain applications, the presence of a fungal skin might be advantageous, for instance, when a more uniform finish is desired. However, achieving a thicker fungal skin may require extended incubation times, allowing for greater development of aerial hyphae on the surface. This extended incubation would increase the environmental costs associated with production.

The successful production of 20 specimens using five different substrates highlights the versatility of *Lentinus tigrinus* in binding a variety of materials. The ability to interchange substrates with minimal physical impact on the final product is a significant advantage for the scalability of mycelium-based composites. This adaptability is particularly beneficial when considering agricultural waste, where substrate availability may vary seasonally or due to other resource fluctuations.

### Thermal performance

**Fig. 4:**
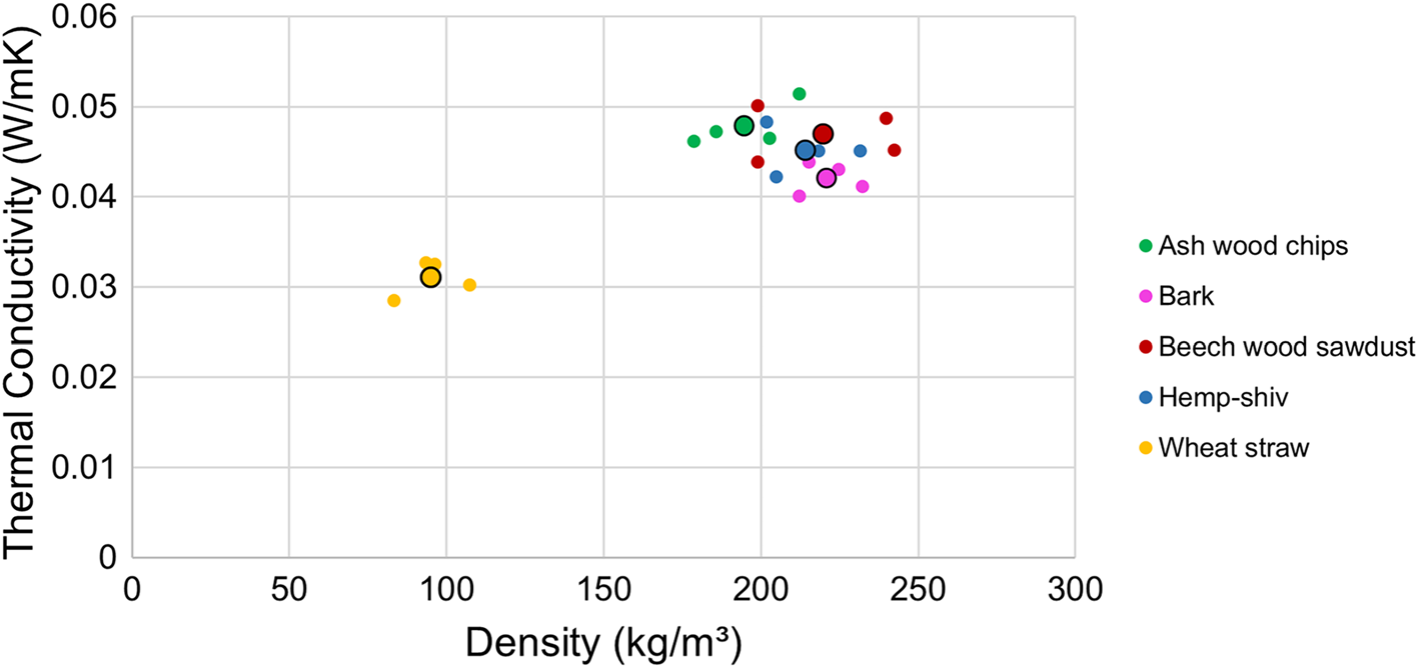
Figure showing the thermal conductivity ($\lambda$) of vs density ($\rho$) of mycelium-based composites made using different substrates, with both individual data points and averages for each substrate plotted.

Figure 4 shows a plot of thermal conductivity ($\lambda$) of the specimens vs density ($\rho$), with both individual data points and averages for each substrate plotted. Table 2 shows the calculated FU based on the results of thermal conductivity and density measurement. All substrates produced MBCs with good insulative properties ($\lambda<$ 0.1 W/m$\cdot$K), comparable to traditional insulation materials. MBCs based on straw displayed the lowest $\lambda$ and the lowest $\rho$ of $0.031 \pm 0.001$ W/m$\cdot$K.

**Table 2:** Table showing the values of $\lambda$ and $\rho$ used in the calculation of the mass of each functional unit (FU) considered in the study.

Substrate	$\lambda$ (W/m$\cdot$K)	$\rho$ (kg/m$^3$)	Mass per FU (kg)
Ash-wood chips	0.048	195	9.31
Bark	0.042	221	9.29
Beech-wood sawdust	0.047	220	10.34
Hemp	0.045	214	9.66
Straw	0.031	129	4.01

The values of $\lambda$ were measured using a heat flow meter according to ASTMC518 and $\rho$ measured according to ASTMC303.

A one-way ANOVA was conducted to determine whether there were statistically significant differences in $\lambda$ among the specimens made using the five substrates. The results revealed a significant difference in $\lambda$ between the specimens made with different substrates ($F = 36.49$, $p = 2.91 \times 10^{-7} < 0.05$), indicating that at least one group differed significantly from the others.

To identify the specific group differences, a Tukey’s Honest Significant Difference (HSD) post-hoc test was performed. This analysis showed that $\lambda$ of specimens made using straw was significantly lower than specimens made with all other tested substrates ($p < 0.05$). Similarly, specimens made using bark exhibited significantly lower ($p < 0.05$) $\lambda$ compared to those made using ash-wood chip and beech-wood sawdust.

However, no statistically significant differences ($p > 0.05$) were found between specimens made using ash-wood chips and beech-wood sawdust, ash-wood chips and hemp shiv, beech-wood sawdust and hemp shiv, or hemp shiv and bark, suggesting that these groups exhibit comparable values of $\lambda$.

A Pearson correlation analysis was conducted to examine the relationship between $\lambda$ and $\rho$. The results revealed a strong positive correlation between the two variables ($r = 0.83$, $p = 1.09 \times 10^{-5} < 0.05$), indicating that as $\rho$ increases, $\lambda$ also tends to increase. The correlation coefficient suggests a strong linear association with the low p-value confirming a statistical significance. The specimens made using straw were then excluded from the data set in a subsequent analysis to see if the notably different properties (lower $\lambda$ and $\rho$) of the straw MBCs were influencing the overall correlation and obscuring trends in the other specimens. After excluding straw based MBCs from the data set, the correlation between $\lambda$ and $\rho$ remains positive ($r = 0.69$) and statistically significant ($p = 0.04 < 0.05$). This suggests an increase in $\lambda$ corresponds to an increase in $\rho$.

These results demonstrate that MBCs with good insulating properties can be made from a diverse array of substrates. Substrates with lower $\rho$ generally exhibited lower $\lambda$, with straw- based substrates showing the lowest values for both. This may be attributed to the high porosity of straw, which can trap insulating pockets of air, of which can lead to the suppression of convection^64^. However, differences in substrate morphology, such as the fibrous nature of straw compared to the particulate forms of other substrates, also likely play a role in influencing heat transfer through the material^65^.

Within individual substrate groups, the relationship between density and thermal conductivity is less clear, suggesting that additional factors, such as the extent and nature of fungal colonisation, contribute to $\lambda$ measurement. Fungal growth may influence the microstructure and connectivity of substrate pores which may influence heat transfer through the material^65,66^.

Moreover, surface contact between the specimen and the heat flow meter may introduce variability and represent a source of uncertainty in measurements, particularly when comparing materials with differences in properties such as orientation^67^. This complicates the identification of definitive trends across a narrow range of $\rho$ and $\lambda$ values.

These results suggest that reducing $\rho$ may improve $\lambda$ to a point, however, further lowering $\rho$ could compromise structural integrity or create issues with uniformity and reproducibility in the material, and this relationship may not be linear. Factors such as mechanical strength, durability, and material handling may limit the extent to which $\rho$ can be reduced without adversely affecting the material’s overall performance. Nonetheless, lighter-weight insulation materials remain attractive due to reduced transportation costs and improved ease of use.

### LCA

In this section, the results of the life cycle assessment of the MBCs produced with different substrates are presented and discussed. The environmental impacts associated with the declared FU of MBC for each substrate are analysed, with a particular focus on comparing Global Warming Potential (GWP) indicators. This focus is due to the construction industry’s significant contribution to global carbon emissions and the increasing emphasis on reducing embodied carbon in building materials. The results of the uncertainty analysis, conducted using Monte Carlo simulations to account for uncertainty in the substrate carbon sequestration factor, are then presented. Additionally, the impact of different transport scenarios on the overall environmental performance of each substrate is examined. Finally, the analysis considers the effect of treating the substrate as a waste product within the LCA framework.

### Substrate comparasion

**Fig. 5:**
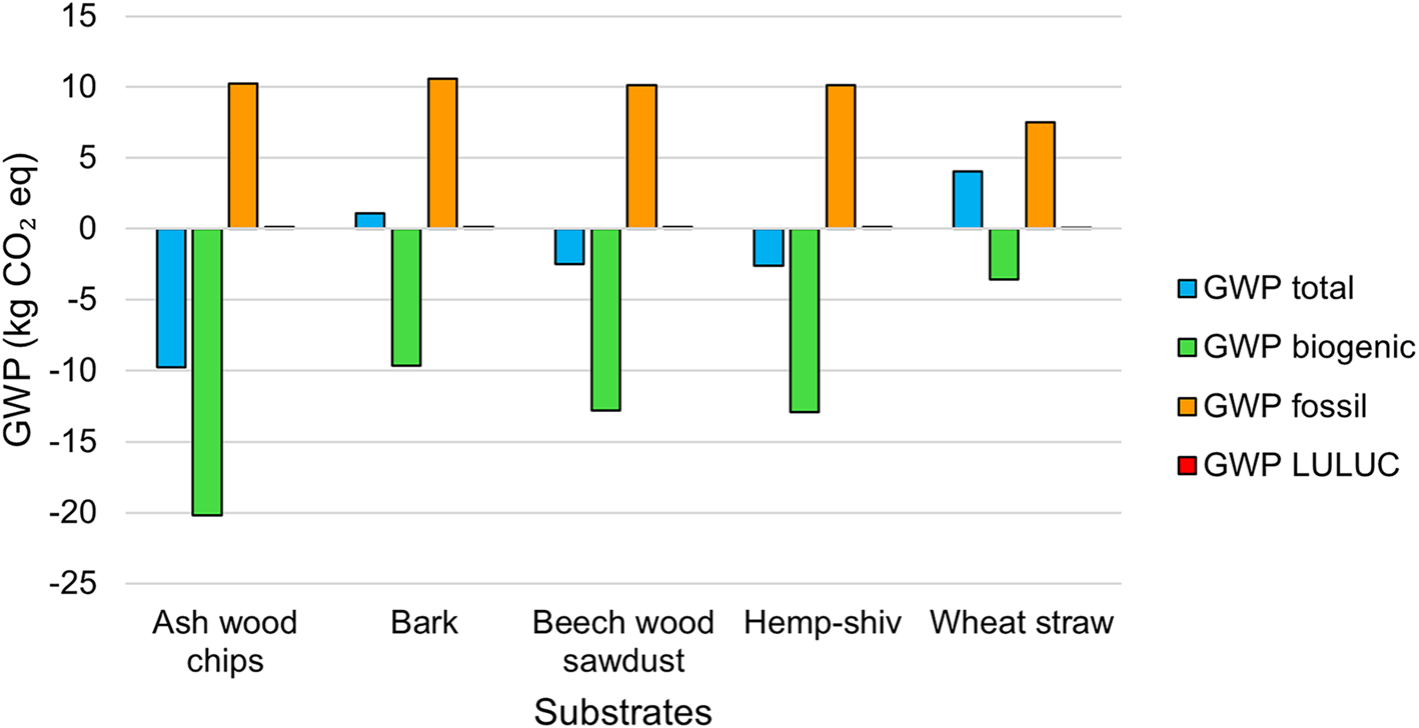
Graph showing the LCA environmental indicators of global warming potential (GWP) (GWP total, GWP biogenic, GWP fossil, GWP land-use-and-land-use-change (LULUC)) for mycelium-based composites produced at a laboratory scale using five different substrates (ash-wood chips, bark, beech-wood sawdust, hemp shiv, wheat straw) using *Lentinus tigrinus* mycelium. The functional unit (FU) of this LCA study is defined as the mass (kg) of MBC insulation required to achieve a thermal resistance (R-value) of 1 ($\hbox {m}^{2}\cdot \hbox {K/W}$) over an area of 1 m$^2$. Under the cradle-to-gate scope adopted in this study, mycelium-based composites made using ash-wood chips demonstrated the lowest GWP total of -9.77 kg CO$_2$ eq.

Table 3 shows the environmental impact per FU of MBC for the five substrates with the transport scenario of 10 km. Fig. 5 shows the GWP indicators (GWP total, GWP biogenic, GWP fossil, GWP land-use-and-land-use-change (LULUC)) for the five different substrate MBCs.

The LCA results for MBCs produced with these five different substrates demonstrate variations in global warming potential (GWP) across the four GWP impact factors. GWP biogenic is associated with carbon sequestration, with ash-wood chips achieving the highest sequestration (-20.16 kg CO$_2$ eq), followed by hemp shiv (-12.89 kg CO$_2$ eq), beech-wood sawdust (-12.78 kg CO$_2$ eq), bark (-9.65 kg CO$_2$ eq), and wheat straw MBCs demonstrated the least carbon sequestration (-3.57 kg CO$_2$ eq). Between the different substrates, GWP fossil showed a lower range of values, ranging between 7.55 kg CO$_2$ eq for straw and 10.25 kg CO$_2$ eq for ash-wood chips. The GWP fossil was attributable to the electricity demands of autoclaving, incubating the MBCs, and drying the MBCs.

For total GWP, which aggregates biogenic, fossil, and LULUC, and under the cradle-to-gate scope adopted in this study, ash-wood chips demonstrated the lowest net emissions (-9.77 kg CO$_2$ eq), suggesting an environmental advantage when considering GWP. Bark exhibited a slight positive emission (1.10 kg CO$_2$ eq), while, like ash-wood chips, beech-wood sawdust-based MBCs and hemp-based MBCs displayed negative total GWP (-2.48 kg CO$_2$ eq and -2.61 kg CO$_2$ eq respectively). Straw- based MBCs showed the highest total GWP of 4.04 kg CO$_2$ eq, making it the least favorable in terms of GWP among the substrates analysed.

Considering the other environmental impact factors, Ozone Depletion Potential (ODP) values are relatively low across all materials, with wheat straw having the lowest value of 7.11$\times 10^{-7}$ kg CFC$_{11}$ eq and ash-wood chips having the highest of 1.06$\times 10^{-6}$ kg CFC$_{11}$ eq. Acidification is highest for bark (0.0828 molc H$^+$ eq) and lowest for wheat straw (0.0506 molc $^+$ eq). The Wood-based substrates (ash-wood chips, bark, and sawdust) showed higher acidification potential than hemp shiv and wheat straw. Eutrophication (EP) is divided into freshwater (EP f), marine (EP m), and terrestrial (EP) eutrophication; across these categories, bark has the highest EP, while wheat straw has the lowest.

Ash-wood chips and bark have the highest values of Photochemical Ozone Formation, (POCP), of 0.0287 kg NMVOC eq and 0.0284 kg NMVOC eq respectively, while wheat straw has the lowest of 0.0185 kg NMVOC eq. This impact, which relates to smog formation, is slightly higher in wood-based substrates, possibly due to the release of volatile organic compounds (VOCs)^68^. Values for resource use of minerals and metals (ADPE) are similar across most materials (around 0.0001 kg Sb eq), with wheat straw being slightly lower (6.87$\times 10^{-5}$ kg Sb eq). Bark (165.9113 MJ) has the highest value for resource use of of fossil fuels (ADPF) while wheat straw (122.9099 MJ) has the lowest, indicating that the bark-based MBC requires more fossil fuel-based energy for processing or transportation compared to other substrates. Furthermore, bark MBCs have the highest water consumption (924.3115 m$^3$), while wheat straw has the lowest (696.8910 m$^3$). Overall, while ash-wood chip MBCs have the lowest total GWP within the scope of this LCA, due to their lower biogenic GWP, they do not perform best across all impact categories. When selecting the best substrate, decision makers must balance these trade-offs based on their priorities (e.g., global warming vs. air quality) and this identifies areas for improvement for MBCs made using different substrates.

**Table 3:** Environmental impact indicators for the conducted LCA, for the mass (kg) of MBC insulation required to achieve a thermal resistance (R-value) of 1 (m$^2$K/W) over an area of 1 m$^2$, in accordance with EN-15804.

Impact category	Ref. unit	Ash-wood chips	Bark	Beech-wood sawdust	Hemp shiv	Wheat straw
GWP- Biogenic	kg CO$_2$ eq	-20.1629	-9.6522	-12.777	-12.8962	-3.57308
GWP- Fossil	kg CO$_2$ eq	10.2499	10.6117	10.1479	10.1404	7.5452
GWP- LULUC	kg CO$_2$ eq	0.1401	0.1389	0.1505	0.1378	0.0690
GWP- Total	kg CO$_2$ eq	-9.7703	1.1012	-2.4760	-2.6154	4.0433
ODP	kg CFC$_{11}$ eq	1.06E-06	9.73E-07	9.60E-07	9.81E-07	7.11E-07
AP	molc H$^+$ eq	0.0692	0.0828	0.0747	0.0723	0.0506
EP f	kg P eq	0.0033	0.0035	0.0035	0.0034	0.0022
EP m	kg N eq	0.0341	0.0468	0.0383	0.0358	0.0267
EP t	molc N eq	0.2318	0.2877	0.2556	0.2435	0.1676
POCP	kg NMVOC eq	0.0287	0.0284	0.0269	0.0268	0.0185
ADPE	kg Sb eq	0.0001	0.0001	0.0001	0.0001	6.87E-05
ADPF	MJ	160.2252	165.9113	157.0042	156.5737	122.9099
WDP	m$^3$	788.5441	924.3115	812.2124	796.2691	696.8910

This analysis was conducted for MBCs produced on a laboratory scale using five different substrates (ash-wood chips, bark, beech-wood sawdust, hemp shiv, and wheat straw). Acronyms: GWP-biogenic (climate change, biogenic), GWP-fossil (climate change, fossil), GWP-LULUC (climate change, land use and land use change), GWP-total (climate change, total), ODP (ozone depletion), AP (acidification), EP f (eutrophication, freshwater), EP m (eutrophication, marine), EP t (eutrophication, terrestrial), POCP (photochemical ozone formation), ADPE (resource use, minerals and metals), and ADPF (resource use, fossils).

**Fig. 6:**
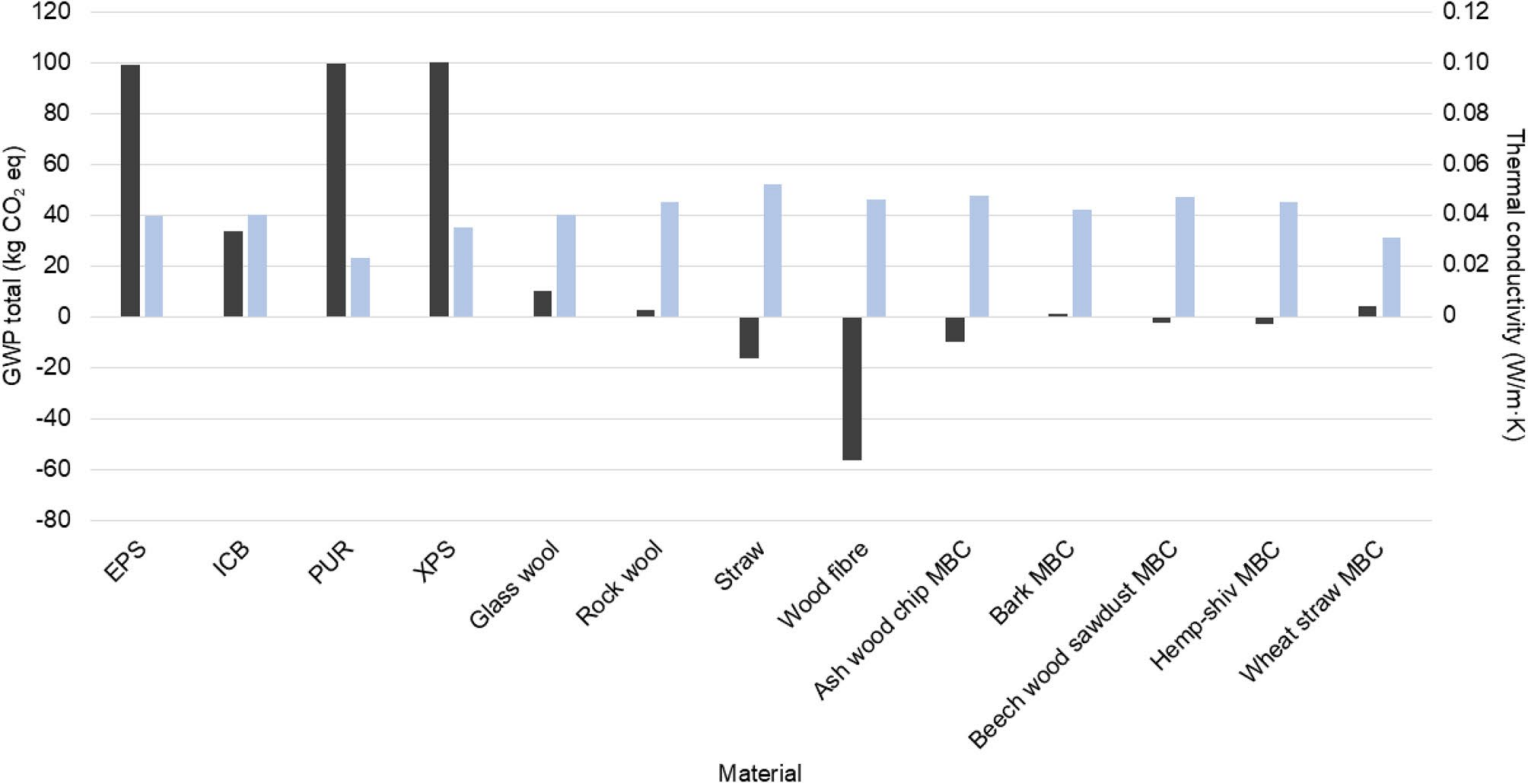
Graph showing the thermal conductivity ($\lambda$) and the total global warming potential (GWP) for the mass (kg) of MBC insulation required to achieve a thermal resistance (R-value) of 1 (m$^2$K/W) over an area of 1 m$^2$ for some traditional insulation materials and the MBC materials produced in this study. Impacts are shown for the A1-A3 stages of the LCA. Acronyms: EPS (expanded polystyrene), ICB (expanded cork agglomerate), PUR (polyurethane), XPS (extruded polystyrene). Data for EPS, ICB, PUR, and XPS were obtained from^52^, data for glass wool and rock wool were sourced from^73^, data for hemp fibre insulation from^74^, and data for straw insulation from^61^.

Figure 6 compares the total GWP per FU of the five MBCs produced in this study with some traditional insulation materials. This comparison highlights the environmental advantages of using MBC insulation solutions compared to certain traditional materials, with all five different MBC insulation materials exhibiting lower total GWP per FU than expanded polystyrene (EPS), expanded cork agglomerate (ICB), polyurethane (PUR), expanded polystyrene (XPS), and glass wool. EPS, PUR, and XPS are associated with particuarly large GWP total, ranging from 99 to 100.1 kg CO$_2$ eq. This reflects the substantial embodied carbon associated with their production. The high GWP of traditional insulaition materials is due to the use of fossil fuels within manufacturing and the feedstock for the plastic resin and blowing agents^69^. Rock wool insulation exhibits a lower GWP total than MBCs in some cases, namely for the wheat straw MBCs, however rock wool insulation. The GWP total values for the MBCs produced in this study are not as low as those reported for straw and hemp fibre insulation materials. This likely reflects, in part, the fact that conventional plant fibre insulation products are manufactured at industrial scale, where process efficiencies and optimised energy use reduce embodied impacts. In addition, MBC production involves several energy intensive stages such as sterilisation, controlled incubation, and oven drying, which are not typically required for minimally processed plant fibre insulation. Improvements in process efficiency, the adoption of lower energy sterilisation methods, shortened incubation periods, or the use of renewable electricity could therefore significantly reduce impacts and help narrow the gap between MBCs and established plant fibre insulation products.

As highlighted in Table 1, the total GWP values reported here are consistent with previous insulation-focused LCAs of MBCs^34,35,38–40^. These studies generally report comparatively low climate change impacts at cradle-to-gate stage. Direct numerical comparison is challenging due to differences in functional units, system boundaries, and biogenic carbon accounting approaches across studies. Nevertheless, the results here are consistent with findings that MBC insulation offers a low-carbon alternative to conventional insulation materials, with further improvements achievable through greater production energy efficiency and that careful consideration of carbon accounting assumptions is needed when comparing results^34,40^.

A related comparison can be drawn with lignocellulosic panels bonded with adhesive resins, such as particleboard and medium-density fibreboard. Recent cradle-to-gate LCAs of resin-bonded panels identify adhesive production, whether urea-formaldehyde, phenol-formaldehyde, polymeric MDI, or alternative bio-based resins, as a significant contributor to fossil GWP and other impact categories, motivating research into lower-impact binder systems and circular feedstocks^70–72^. In contrast, MBCs utilise fungal mycelium as a natural adhesive, eliminating the need for synthetic adhesive resins. While these materials are not functionally equivalent to structural wood panels and therefore cannot be directly compared on the present thermal-performance functional unit, the absence of petrochemical binders represents an important component of material design that contributes to maintaining low fossil GWP. This difference may reduce reliance on fossil-derived inputs in comparison to conventional resin-bonded systems, although mechanical performance, durability, and fire resistance requirements must be considered when evaluating substitution potential.

The total GWP results will be influenced by the distinction between biogenic and fossil carbon flows and by the adopted cradle-to-gate system boundary. Under the EN 15804 static -1/+1 accounting approach, biogenic carbon uptake during biomass growth is credited at the production stage (A1-A3), while potential carbon release at end-of-life is not included within the current scope. As a result, substrates with higher carbon content per functional unit benefit from larger temporary carbon storage, which reduces total GWP in a cradle-to-gate assessment. However, if the system boundary were extended to include end-of-life processes (C1-C4), and full oxidation of biogenic carbon were assumed through combustion or biodegradation, the net GWP values would increase accordingly. Under such assumptions, total GWP would be driven primarily by fossil and land-use related emissions, potentially favouring substrates such as wheat straw that require lower mass per functional unit and therefore exhibit lower fossil GWP. Furthermore, end-of-life treatment pathways for bio-based insulation materials remain uncertain, with outcomes ranging from composting and landfill storage to energy recovery, each associated with different carbon release profiles and timing. Dynamic approaches to global warming potential, which account for the temporal delay between carbon uptake and release, may therefore provide additional insight into the environmental impact of MBCs and further research is needed into the end-of-life stages of these materials. The results of this study should consequently be interpreted within the defined cradle-to-gate scope, recognising that substrate ranking is sensitive to end-of-life assumptions and carbon accounting methodology.

### Carbon sequestration uncertainty

The LCA results suggest that the total GWP is strongly influenced by the biogenic GWP, which in turn depends on the carbon sequestration factor of the substrate. The carbon sequestration factor, sourced from literature, carries inherent uncertainty due to variations in species composition, growth conditions, and biological characteristics of the material. For a given material, the higher it’s carbon sequestration potential the better as this means it removes more carbon dioxide from the environment^75–77^. Given its impact on total GWP estimates, it is important to consider how this uncertainty affects the LCA outcomes and, consequently, the selection of the most suitable substrates.

**Fig. 7:**
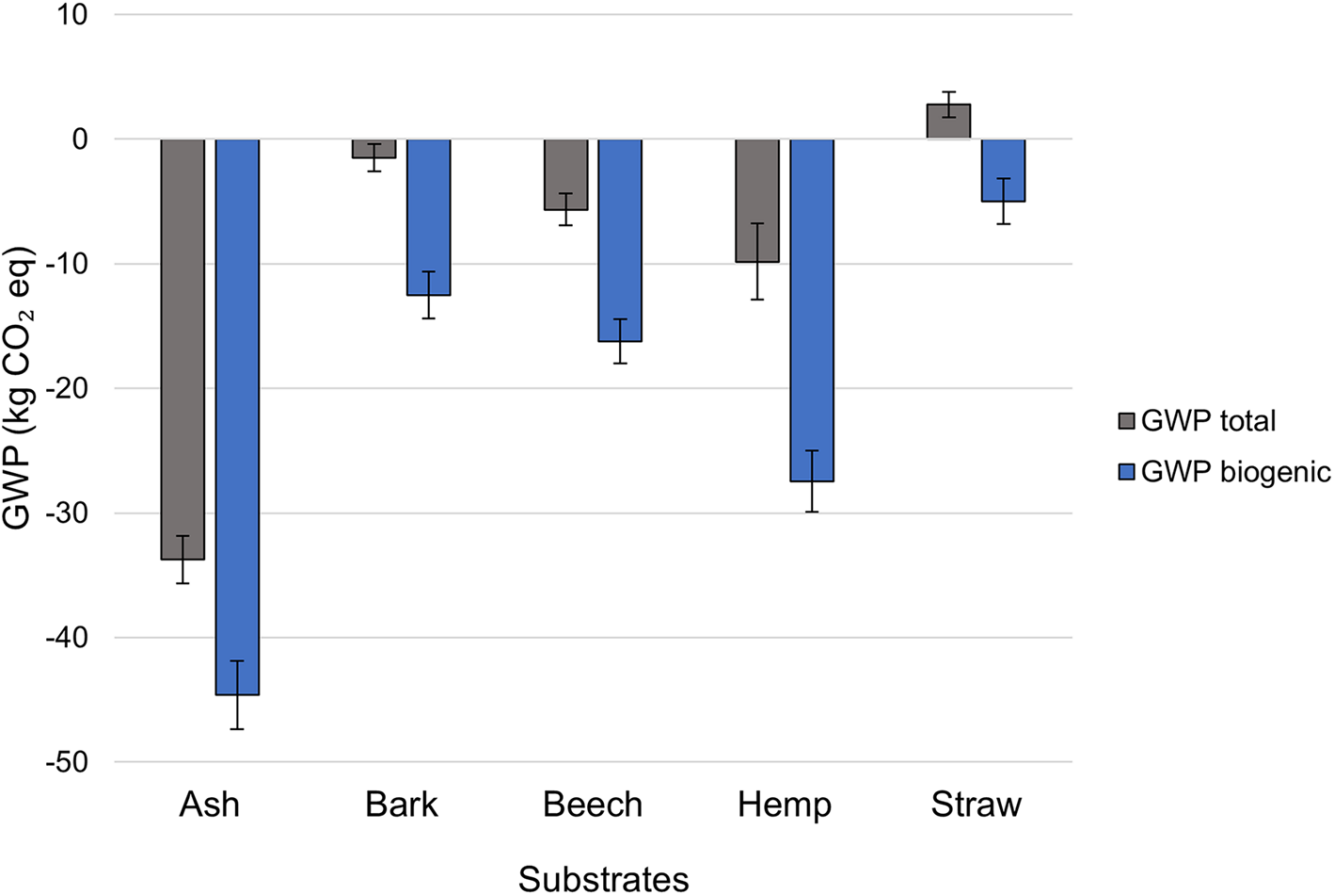
Monte Carlo simulation results for the total GWP and biogenic GWP of MBCs made with five different substrates. The bar chart presents the mean total GWP and mean biogenic GWP for each substrate after 10000 Monte Carlo iterations with the carbon sequestration factor varying by 1 standard deviation in the input. The bars on the chart represent the standard deviation in mean GWP value obtained. Standard error in the mean was between 0.01 and 0.04 for these values.

To evaluate the influence of this variability, a Monte Carlo simulation was performed as detailed in the methods section. This approach allows for a probabilistic assessment of the results, accounting for the range of possible values associated with the sequestration factor. Fig. 7 presents the simulation results, displaying the mean total GWP after 10000 iterations, with error bars representing the standard deviation. Table 4 details the mean value, standard deviation, 5th and 95th percentile, and the median value of the simulations.

**Table 4:** Monte Carlo simulation results for the total GWP and biogenic GWP of MBCs made with five different substrates.

Material	Mean	Std. deviation	5th percentile	95th percentile	Median
GWP Total (kg CO$_2$ eq)					
Ash-wood chip	−33.73	2.74	−37.31	−30.37	−33.84
Bark	−1.49	1.90	−3.59	0.59	−1.58
Beech-wood sawdust	−5.65	1.77	−7.99	−3.46	−5.67
Hemp shiv	−9.84	2.45	−13.93	−5.99	−9.79
Straw	2.78	1.83	0.91	4.60	2.74
GWP Biogenic (kg CO$_2$ eq)					
Ash-wood chip	−44.62	1.91	−47.91	−41.62	−44.50
Bark	−12.53	1.11	−14.37	−10.70	−12.50
Beech-wood sawdust	−16.21	1.26	−18.32	−14.23	−16.17
Hemp shiv	−27.44	3.06	−32.77	−22.72	−27.26
Straw	−4.99	1.01	−6.68	−3.33	−4.97

The table presents the mean, standard deviation, 5th percentile, 95th percentile, and median after 10000 Monte Carlo iterations, with the carbon sequestration factor varying by one standard deviation in the input.

This Monte Carlo analysis provides several insights into the variability and uncertainty of their biogenic and total GWP, and indicate that the carbon sequestration ability influences the overall uncertainty, making it an important parameter for further consideration in LCAs. The result indicate a sensitivity of GWP results to the uncertainty in carbon sequestration. With an input standard deviation of 1, the resulting variability in the outputs exceeds 1 in all cases. In particular, the total GWP of ash wood chip based MBC, as well as the biogenic GWP of both ash wood chip based and hemp based MBCs, show standard deviations greater than 2. This suggests that small variations in carbon sequestration can lead to relatively large changes in GWP estimates, highlighting the need for improved data on this parameter. However, the uncertainty in carbon sequestration is partly biological in nature and is affected by growth conditions and plant species, and thus some level of variability is unavoidable. It is therefore important to assess how this variability impacts material ranking and decision-making.

Although the results indicate a sensitivity to carbon sequestration, the results also indicate that the assumption of normality in the Monte Carlo model is reasonable, as the mean and median values are similar across the 5 MBCs. This suggests that the distributions are symmetric and that the extreme values are not skewing the results.

Considering some key results, it can be seen that the bark-based MBC can, in some cases, shift from acting as a net carbon sink to a slight net emitter. While the overall impact remains small, this finding underscores the importance of considering uncertainty in material assessments, particularly for substrates with lower carbon storage potential.

Consider the deterministic LCA conducted in this study, ash-wood chip-based MBC has the lowest total GWP, making it the most favourable material. However, when considering uncertainty, the ranking of materials with closer GWP values (bark, beech-wood sawdust, and hemp shiv) could change under certain scenarios. While this suggests that some substrates may perform differently depending on the assumptions used, the standard error in the mean GWP does not overlap between these materials, providing some confidence in their relative ranking.

Overall, this uncertainty analysis highlights the importance of incorporating uncertainty assessments in MBC sustainability studies, as deterministic approaches alone may not fully capture the range of possible outcomes. Future research should aim to improve estimates of the carbon sequestration of a material, assess the impact of biological variability, and explore the conditions under which material ranking may shift. A more complete uncertainty analysis could be undertaken to account for additional uncertainties in input data, such as energy use and other process-related factors. However, since this LCA was conducted for the laboratory-scale production of these materials, it is important to acknowledge that energy consumption and other inputs will likely change as the process scales up or undergoes modifications. As a result, the primary focus of uncertainty assessment in this study was on the raw substrate, as its impact is expected to remain more consistent across different production scenarios. Furthermore, the main MBC production steps (e.g. the stages shown in Fig. 1) are the same for all substrates and assessing uncertainty in the substrate specific stages is more informative when considering the study goals. Understanding these uncertainties will help in making more informed decisions about the environmental performance of mycelium-based composites.

### Transport

**Fig. 8:**
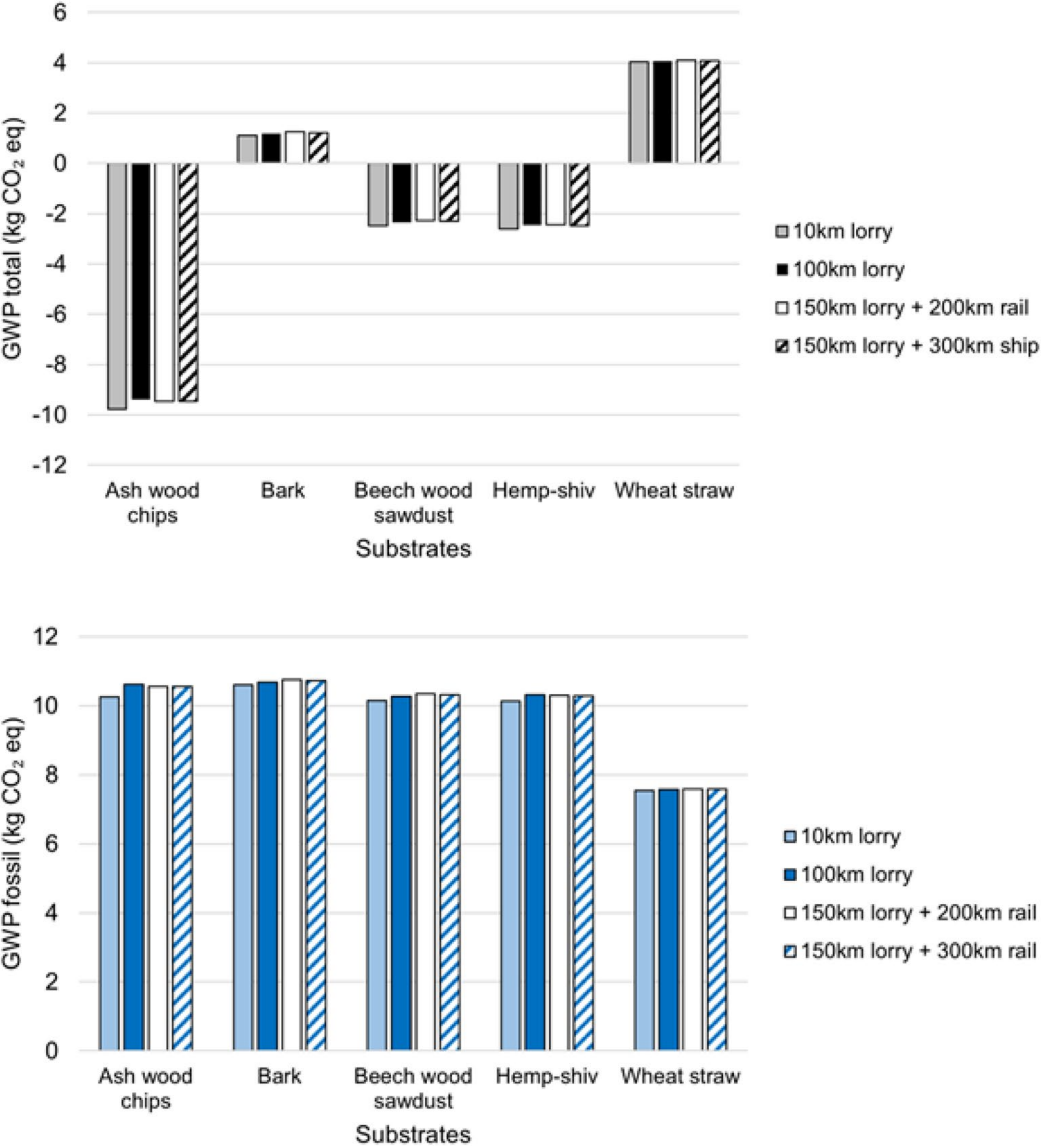
Figure showing (**a**) the GWP total and (**b**) the GWP fossil environmental impacts for producing mycelium-based composites using five different substrates (ash-wood chips, bark, beech-wood sawdust, hemp shiv, wheat straw) with four different scenarios considered for the transport of the raw substrate to the MBC production location. The functional unit (FU) of this LCA study is defined as the mass (kg) of MBC insulation required to achieve a thermal resistance (R-value) of 1$^2$K/W over an area of 1 m$^2$.

Fig. 8 illustrates the a) GWP total and b) GWP fossil for producing a FU of the MBCs produced using each of the five different substrates considered, comparing four transport (of the raw substrate to the location of MBC production) scenarios.

The LCA results for mycelium-based composites produced with the five different substrates under the four transport scenarios demonstrate that substrate selection remains the dominant factor influencing the overall GWP impact. However, transport scenarios do introduce some variations in GWP impact and for a given substrate should be minimised where possible. In Scenario 1 (10 km freight), ash-wood chip exhibits the lowest total GWP (-9.77 kg CO$_2$ eq), followed by hemp shiv (-2.62 kg CO$_2$ eq) and beech-wood sawdust (-2.48 kg CO$_2$ eq). Straw, with 4.04 kg CO$_2$ eq was the least favorable considering this indicator.

As the transport distance increases to 100 km freight (Scenario 2), relatively small increases in GWP were observed. Ash-wood chip remains the substrate with the lowest GWP with -9.40 kg CO$_2$ eq and straw is still associated with the highest total GWP of 4.07 kg CO$_2$ eq. Scenario 3 (150 km freight plus 200 km rail) and Scenario 4 (150 km freight plus 300 km ship) introduce higher GWP values for all substrates, but ash-wood chip remains the most favourable (-9.46 and -9.47 kg CO$_2$ eq, respectively). Across all scenarios, bark and straw consistently rank as less optimal with the highest total GWP values.

Despite transport contributing to increased GWP across the board, in this analysis the inherent properties of the substrates drive the overall environmental performance.

### Waste vs non-waste

**Fig. 9:**
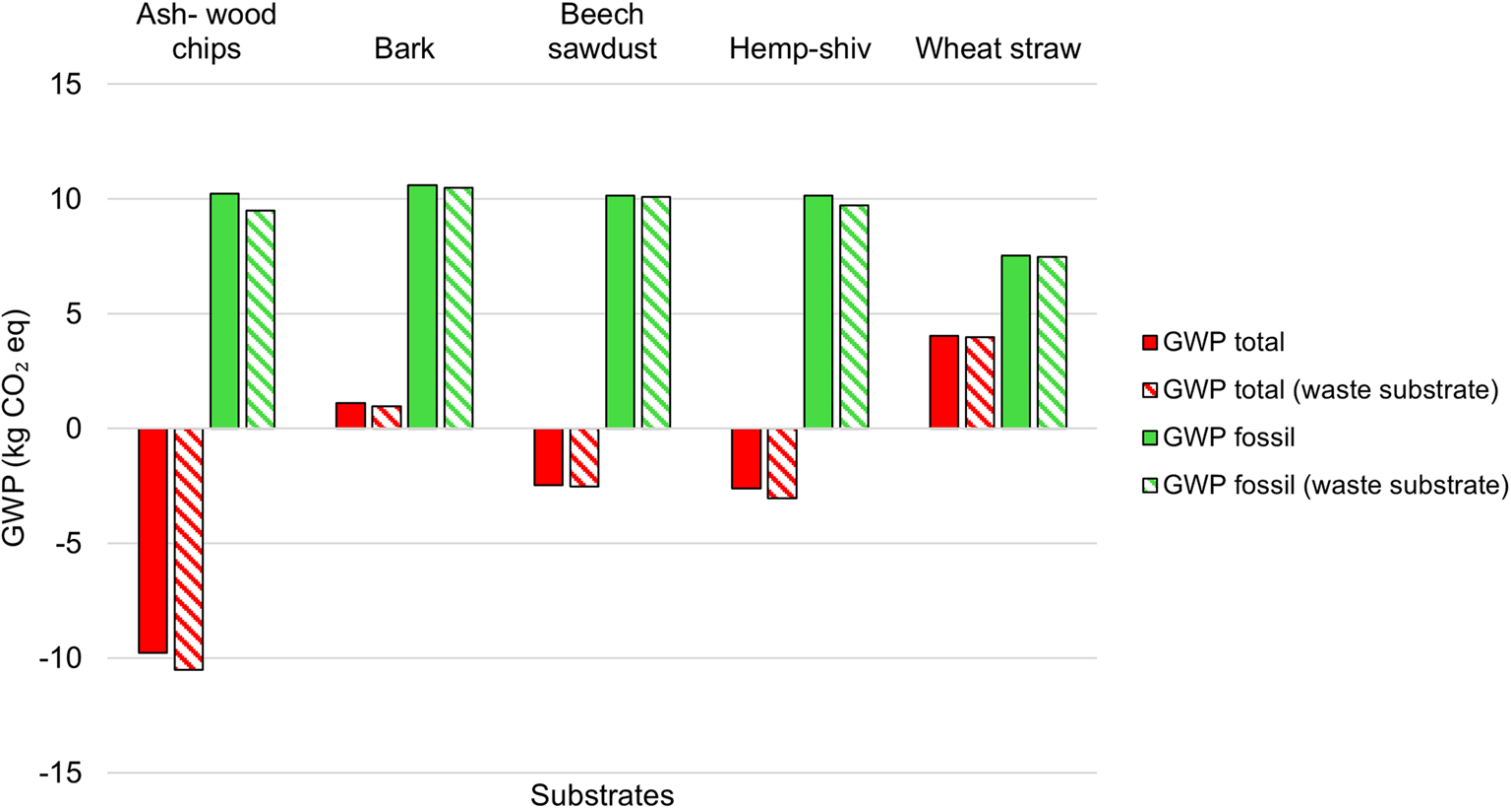
GWP impacts for total GWP and fossil GWP for the five different MBCs when the substrate (ash-wood chips, bark, beech-wood sawdust, hemp shiv, and wheat straw) is designated as waste or not waste.

Figure 9 shows the GWP total and GWP fossil for the five different MBCs when the substrate is designated as a waste or as a non-waste. In all scenarios, treating the agricultural substrate as a pure waste results in a lower GWP. However, the differences in GWP are relatively small, and do not affect the relative impacts between the substrate and therefore substrate selection. The fossil GWP differences are as follows: 7.3% for ash-wood chips, 1.1% for bark, 0.5% for beech-wood sawdust, 4.0% for hemp shiv, and 1.0% for wheat straw. These minimal differences may be attributed to the fact that the impacts associated with obtaining these agricultural materials when not treated as waste are relatively low per kilogram of material. This is particularly true when compared to the production of more energy-intensive materials like plastics and steel. The production of agricultural materials typically involves mechanical processes that do not require high temperatures or chemical treatments, which contributes to their lower overall environmental impact. Future work could investigate how the environmental impact of the MBCs changes when materials are sourced from unsustainable agricultural systems. Factors such as intensive fertiliser use and agriculture associated with deforestation could increase the overall GWP of the MBCs. In particular, deforestation linked agricultural practices could lead to higher LULUC GWP, thereby increasing the broader environmental impact of the materials^78^. The relatively small impact of waste designation on GWP is an important consideration, as it can alleviate the pressure of making this decision, which is not always straightforward. Many of these materials are byproducts or waste products that have alternative uses, such as pet bedding or biofuel, making the classification less clear-cut.

Although designating the substrate as waste resulted in relatively small changes in total GWP, with percentage reductions ranging from 1.75% for straw to 14% for bark, other impact categories were more strongly affected. In particular, acidification decreased by between 247% and 251% across all substrates, with a notably consistent reduction. Acidification potential reflects emissions that contribute to soil and water acidification, primarily from substances such as sulphur dioxide, nitrogen oxides, and ammonia, which can damage ecosystems and alter soil chemistry. This suggests that waste designation may be especially important when acidification is considered a key environmental indicator.

Other categories also showed variation. ADPE changed by between 1% for bark and beech wood sawdust and 16% for hemp shiv, while photochemical ozone formation changed by 1% for beech wood sawdust and straw and up to 16% for ash. Freshwater eutrophication varied by as much as 4% for ash wood.

Overall, while total GWP is not substantially affected by waste designation, other environmental indicators appear to be more sensitive. This highlights the importance of considering the full set of impact categories when evaluating waste assumptions and associated uncertainties. It also indicates that modelling substrates as pure waste can reduce impacts across multiple categories, particularly acidification, although these large percentage changes should be interpreted in the context of relatively low initial impact values.

### LCA summary

The LCA conducted revealed a range of environmental impacts and GWP depending on the substrate used, with the material choice being the most influential factor compared to transport distance and waste designation. Comparing the five different substrates, ash-wood chips, bark, beech-wood sawdust, hemp shiv, and wheat straw, it was found the best case scenario in terms of GWP corresponds to an MBC made using locally source ash-wood chips which are designated as waste products, where as the worst case scenario is to use wheat-straw sourced non-locally and not considered a waste material. Focusing on carbon sequestration, the uncertainty analysis focused on carbon sequestration using Monte Carlo simulations revealed that the ranking of materials remains generally consistent, although some substrates (e.g. the bark-based MBCs) may fluctuate between acting as a net carbon sink or a slight net emitter when subject to uncertainty in the carbon sequestration. In all scenarios considered, the GWP of the MBCs is relatively low compared to traditional insulation materials.

### Limitations and scope considerations

This study is subject to limitations inherent to both the system boundary and the laboratory-scale production context. While the LCA includes land use and land-use change (LULUC) indicators as defined within EN 15804, indirect land-use change and broader system-level effects associated with increased biomass demand were not modelled. The potential competition between biomass feedstocks and food production, particularly for agricultural residues such as wheat straw and hemp shiv, was therefore not explicitly assessed. Although the materials considered are typically classified as residues or by-products, increased demand for such feedstocks could influence land management practices and crop allocation in ways not captured in a cradle-to-gate attributional LCA.

Furthermore, the assessment adopts a static biogenic carbon accounting approach consistent with EN 15804, crediting carbon uptake during biomass growth without modelling the temporal dynamics of carbon release at end-of-life. MBCs may act as temporary carbon stores during their service life; however, the magnitude and timing of carbon release depend on disposal pathways, which may include composting, landfill storage, energy recovery, or incineration. These pathways involve different carbon release rates and climate implications, and were not explicitly modelled in this study. As a result, conclusions regarding total GWP are sensitive to end-of-life assumptions and carbon accounting methodology.

Additionally, the LCA is based on laboratory-scale production data. Energy use for sterilisation, incubation, and drying at small scale is typically less efficient than industrial-scale processes, and scaling up production may alter the relative contribution of energy inputs to total impacts^34^. Conversely, industrial production could introduce additional impacts associated with infrastructure or modified growth conditions^79^. The results presented here therefore reflect comparative performance under laboratory conditions and should not be interpreted as definitive industrial-scale environmental performance. Nevertheless, one potential advantage of MBCs lies in their relatively simple production process and low barrier to entry, suggesting that small-scale or decentralised manufacturing may represent a realistic and relevant application pathway for these materials^80^.

Overall, the findings provide insight into substrate-driven differences under defined assumptions but should be interpreted within the constraints of system boundary, allocation methodology, and production scale.

## Conclusion

This study aimed to assess the thermal performance of mycelium-based composites produced from different substrates and to evaluate their overall environmental impact using life cycle assessment. Five MBCs were produced and thermally characterised to determine the functional unit for the LCA. In addition to assessing the direct environmental effects of substrate selection, the study considered how factors such as transport distances and substrate classification (waste vs. non-waste) influence environmental impacts. The study also investigated the impact of uncertainty in carbon sequestration associated with different substrates.

Since the LCA was conducted at a laboratory scale, the results are subject to change as production processes scale up and as variability arises between research settings. Despite this, the study provides valuable insights into how MBCs can be optimised and highlights directions for future research. The key conclusions of the study are as follows:

- The findings demonstrate that a variety of organic materials can be successfully used to produce MBCs, with the production process remaining similar for the substrate used in this study. This is important for adapting to the availability of raw materials whilst still maintaining similar production processes.
- All tested substrates produced MBCs with good thermal insulation properties, with thermal conductivities below 0.05 W/m$\cdot$K. Among them, the straw-based MBCs exhibited the lowest thermal conductivity of $0.031 \pm 0.001$ W/m$\cdot$K. These values are comparable to other MBCs as well as to traditional insulation materials.
- The LCA results showed that MBCs have low GWP, with several materials having a negative total GWP. The environmental performance of MBCs was found to be comparable to, or better than, other MBCs and to conventional insulation materials. However, despite having the best thermal conductivity, straw-based MBCs performed the worst in terms of total GWP. This was attributed to their lower carbon sequestration compared to other substrates. This finding highlights the need for research efforts to balance thermal conductivity optimisation with environmental impact rather than prioritising insulation performance alone.
- The results suggest that small differences in thermal performance do not significantly impact the overall GWP outcomes. This is important as thermal characterisation of bio-based materials can be subject to uncertainties due to their anisotropic nature and poor surface contact in measurements. The results emphasises that minor improvements in insulation performance should not be prioritised over broader environmental considerations.
- The impact of transport distances on GWP was found to be relatively small. Although sourcing a substrate from a closer location does reduce GWP, this difference was not large enough to affect the overall ranking of substrates. For example, an MBC produced from ash-wood chips sourced from a distant location still had a lower GWP than a locally sourced straw-based MBC. This suggests that substrate selection should focus more on material properties rather than transport distances, though factors such as cost and ease of procurement may also be important for decision-making.
- The study found that GWP is sensitive to the carbon sequestration of the substrate. While this factor must be considered, some degree of uncertainty is unavoidable due to the biological nature of the materials. Despite this variability, the overall ranking of materials in this study remained consistent.
- The classification of agricultural waste as either a waste or non-waste material had a small effect on total GWP, with a lower GWP obtained when the substrate is designated a waste. The fact this difference is small is likely because the preparation of these agricultural materials has a relatively low environmental burden.

Overall, this study highlights the versatility of MBCs and their ability to be produced from a range of substrates while maintaining strong thermal and environmental performance. The findings suggest that the most effective way to reduce the environmental impact of MBCs is to focus on reducing energy demands during production rather than attempting to optimise insulation performance through substrate selection. For MBCs to be viable insulation materials, future research should evaluate their durability, incorporate this into the functional unit in LCAs, and investigate the scalability of MBCs with a diverse range of substrate feedstocks.

## Supplementary Information

Supplementary Information.

## Data Availability

The datasets generated during and/or analysed during the current study are available from the corresponding author on reasonable request.
