# Supplementary material for: Comparing substrates for mycelium-based composite insulation materials with thermal and environmental assessment
Source: Sci Rep. 2026 Apr 14;16:17466. doi: 10.1038/s41598-026-48045-w (PMC13237016; doi:10.1038/s41598-026-48045-w)
Supplement: Supplementary file 1 — Supplementary Information. [file 41598_2026_48045_MOESM1_ESM.docx]

**Supplementary Information (SI) for:**

**Comparing Substrates for Mycelium-Based Composite Insulation Materials with Thermal and Environmental Assessment**

Joni Wildman^1*^, Valeria Cascione^2^, Daniel Henk^3^, Andrew Shea^1^

^1^Department of Architecture and Civil Engineering, University of Bath, Bath, UK, BA2 7AY

^2^Institute of Sustainability and Climate Change, University of Bath, Bath, UK, BA2 7AY

^3^The Milner Centre for Evolution, University of Bath, Bath, UK, BA2 7AY

*jlw89@bath.ac.uk

Life Cycle Inventory Ash MBC:

| Process | Item | Quantity | Unit | Data source process | Data source quantity |
| --- | --- | --- | --- | --- | --- |
| Substrate preparation | Wood chips | 12.44 | kg | Ecoinvent v3.6  (UUID = 7fe99768-d571-4bc2-a272-7df585bd0d48) | On-site measurement |
|  | Transport | 2.49 | Tonnes * kg | Ecoinvent v3.6  (UUID = f4e8b38b-d6c7-423b-bc9c-13b4a13d55f8) | Assume distance of 10km with a 16 tonne lorry filled with wood chips to the payload of 10 tonnes |
|  | Carbon | 16.09 | kg | Ecoinvent v3.6  (UUID = cc6a1abb-b123-4ca6-8f16-38209df609be) | Biogenic carbon accounting follows the EN 16485:2023 guidelines[1]. The biogenic carbon stored in the ash wood was calculated following the EN  16449:201448 standard [2], according to the formula: Pco2=4412×cf×m1+w100  , where Pco2 is the biogenic carbon storage in the wood (kg  CO2 eq), cf is the carbon fractional content of the wood [3,4], m is the mass of the wood, and w is the moisture  content of the ash wood. |
| Substrate soaking | Water | 42.93 | L | Ecoinvent v3.6 (UUID = c5adb1fb-872e-4446-a3bb-c4b61aa4bd45) | On-site measurement |
|  | Wood chips | 12.44 | kg | From substrate preparation | On-site measurement |
| Substrate sterilisation | Soaked wood chips | 21.46 | kg | From substrate soaking | On-site measurement (73% increase in mass of substrate upon soaking) |
|  | Electricity | 6.70 | MJ | Ecoinvent v3.6 (UUID = 759b89bd-3aa6-42ad-b767-5bb9ef5d331d) | Energy use for autoclave adapted from Garcia et al. 2012 [8] |
|  | Water | 7.45 | L | Ecoinvent v3.6 (UUID = c5adb1fb-872e-4446-a3bb-c4b61aa4bd45) | Water use for autoclave adapted from Garcia et al. 2012 [8] |
|  | Wheat flour | 2.38 | kg | Ecoinvent v3.6 (UUID = c7b1f264-abe2-47c4-8fcf-db3229228485) | On-site measurement |
|  | Polypropylene granulate | 0.09 | kg | Ecoinvent v3.6 (UUID = 66ca2f38-5e51-4546-83c0-d7cef0c55c7c) | Modelling an autoclavable bag  On-site measurement of 8kg of substrate per bag |
| Grain spawn preparation | Rice | 0.66 | kg | Ecoinvent v3.6 (UUID = 922db745-4df5-4b82-bbb6-98e81e4007d1) | On-site measurement (dry rice to cooked rice ratio 75:225) |
|  | Water | 1.32 | L | Ecoinvent v3.6 (UUID = c5adb1fb-872e-4446-a3bb-c4b61aa4bd45) | On-site measurement (water required for rice cooking) |
|  | Electricity | 0.86 | MJ | Ecoinvent v3.6 (UUID = 759b89bd-3aa6-42ad-b767-5bb9ef5d331d) | Electricity required for rice cooking, using q=mcΔT, temperature change 80˚C to boil water, simmer for 20 mins with a 250W heater, then E=Pt |
|  | Water | 0.83 | L | Ecoinvent v3.6 (UUID = c5adb1fb-872e-4446-a3bb-c4b61aa4bd45) | Water use for autoclave adapted from Garcia et al. 2012 [8] |
|  | Electricity | 4.84 | MJ | Ecoinvent v3.6 (UUID = 759b89bd-3aa6-42ad-b767-5bb9ef5d331d) | Energy use for autoclave adapted from Garcia et al. 2012 [8] |
|  | Polypropylene, granulate | 0.03 | kg | Ecoinvent v3.6 (UUID = 66ca2f38-5e51-4546-83c0-d7cef0c55c7c) | Modelling an autoclavable bag  On-site measurement of 8kg of substrate per bag |
|  | Latex | 0.002 | kg | Ecoinvent v3.6 (UUID = b0cd6c3d-d1b6-42f4-8e58-ca6d4d6c3e18) | Modelling nitrile gloves  On-site measurement of 1 pair |
|  | Water | 0.038 | L | Ecoinvent v3.6 (UUID = c5adb1fb-872e-4446-a3bb-c4b61aa4bd45) | On-site measurement (water to make 70% alcohol spray) |
|  | Ethanol | 0.088 | L | Ecoinvent v3.6 (UUID = 3a0d77a5-6488-4977-966a-731350be864a) | On-site measurement (ethanol to make 70% alcohol spray) |
|  | Electricity | 0.1 | MJ | Ecoinvent v3.6 (UUID = 759b89bd-3aa6-42ad-b767-5bb9ef5d331d) | Energy use of laminar flow hood, data from laminar flow hood technical data sheet [9] |
|  | Electricity | 2.05 | MJ | Ecoinvent v3.6 (UUID = 759b89bd-3aa6-42ad-b767-5bb9ef5d331d) | Energy use of lab incubator, data from laminar flow hood technical data sheet [10] |
| Mixing | Grain spawn | 2.65 | kg | Inoculated grain from grain spawn preparation | On-site measurement |
|  | Sterile substrate | 23.84 | kg | Sterilised substrate from substrate sterilisation | On-site measurement |
|  | Polyethylene, high density | 0.02 | kg | Ecoinvent v3.6 (UUID = b685192a-e033-421e-9074-8fe7cb176046) | Modelling plastic reusable mould, 1.2 square meters of HDPE with a thickness of 1 mm, used 50 times |
|  | Polyethylene, low density | 0.01 | kg | Ecoinvent v3.6 (UUID = 947a85fa-114e-4d9e-b36c-3293e48ea5ae) | Modelling parafilm sheet over the mould, 2 square meters |
|  | Ethanol | 0.07 | L | Ecoinvent v3.6 (UUID = 3a0d77a5-6488-4977-966a-731350be864a) | On-site measurement (ethanol to make 70% alcohol spray) |
|  | Water | 0.03 | L | Ecoinvent v3.6 (UUID = c5adb1fb-872e-4446-a3bb-c4b61aa4bd45) | On-site measurement (water to make 70% alcohol spray) |
|  | Latex | 0.002 | kg | Ecoinvent v3.6 (UUID = b0cd6c3d-d1b6-42f4-8e58-ca6d4d6c3e18) | Modelling nitrile gloves  On-site measurement of 1 pair |
|  | Electricity | 0.24 | MJ | Ecoinvent v3.6 (UUID = 759b89bd-3aa6-42ad-b767-5bb9ef5d331d) | Energy use of laminar flow hood, data from laminar flow hood technical data sheet [9] |
| Incubation | Mixed composite | 26.50 | kg | Mixed composite from mixing stage | On-site measurement |
|  | Electricity | 22.04 | MJ | Ecoinvent v3.6 (UUID = 759b89bd-3aa6-42ad-b767-5bb9ef5d331d) | Energy use of lab incubator, data from laminar flow hood technical data sheet [10] |
| Drying | Colonised composite | 26.50 | kg | Colonised composite following incubation | On-site measurement |
|  | Electricity | 25.9 | MJ | Ecoinvent v3.6 (UUID = 759b89bd-3aa6-42ad-b767-5bb9ef5d331d) | Energy use of laboratory oven, data from laboratory oven technical data sheet [11] |

*Table 1: Life Cycle Inventory: Life cycle inventory for the laboratory-scale production of 1 kg of mycelium-based composites (MBCs) using ash wood chip as a substrate for the scenario where the substrate is transported 10km. Inputs are categorised by process stages, with quantities, units, and data sources specified. Where possible, inputs specific to the GB market were selected for the life cycle inventory.*

Life Cycle Inventory Bark MBC:

| Process | Item | Quantity | Unit | Data source process | Data source quantity |
| --- | --- | --- | --- | --- | --- |
| Substrate preparation | Bark | 3.95 | kg | Ecoinvent v3.6  (UUID = 7fe99768-d571-4bc2-a272-7df585bd0d48) | On-site measurement |
|  | Transport | 0.79 | Tonnes * kg | Ecoinvent v3.6  (UUID = f4e8b38b-d6c7-423b-bc9c-13b4a13d55f8) | Assume distance of 10km with a 16 tonne lorry filled with wood chips to the payload of 10 tonnes |
|  | Carbon | 6.91 | kg | Ecoinvent v3.6  (UUID = cc6a1abb-b123-4ca6-8f16-38209df609be) | Biogenic carbon accounting follows the EN 16485:2023 guidelines[1]. The biogenic carbon stored in the bark was calculated following the EN  16449:201448 standard [2], according to the formula: Pco2=4412×cf×m1+w100  , where Pco2 is the biogenic carbon storage in the bark (kg  CO2 eq), cf is the carbon fractional content of the bark OSB[3,5], m is the mass of the bark, and w is the moisture  content of the bark. |
| Substrate soaking | Water | 40.90 | L | Ecoinvent v3.6 (UUID = c5adb1fb-872e-4446-a3bb-c4b61aa4bd45) | On-site measurement |
|  | Bark | 3.95 | kg | From substrate preparation | On-site measurement |
| Substrate sterilisation | Soaked bark | 20.45 | kg | From substrate soaking | On-site measurement (73% increase in mass of substrate upon soaking) |
|  | Electricity | 6.69 | MJ | Ecoinvent v3.6 (UUID = 759b89bd-3aa6-42ad-b767-5bb9ef5d331d) | Energy use for autoclave adapted from Garcia et al. 2012 [8] |
|  | Water | 7.43 | L | Ecoinvent v3.6 (UUID = c5adb1fb-872e-4446-a3bb-c4b61aa4bd45) | Water use for autoclave adapted from Garcia et al. 2012 [8] |
|  | Wheat flour | 2.27 | kg | Ecoinvent v3.6 (UUID = c7b1f264-abe2-47c4-8fcf-db3229228485) | On-site measurement |
|  | Polypropylene granulate | 0.09 | kg | Ecoinvent v3.6 (UUID = 66ca2f38-5e51-4546-83c0-d7cef0c55c7c) | Modelling an autoclavable bag  On-site measurement of 8kg of substrate per bag |
| Grain spawn preparation | Rice | 0.63 | kg | Ecoinvent v3.6 (UUID = 922db745-4df5-4b82-bbb6-98e81e4007d1) | On-site measurement (dry rice to cooked rice ratio 75:225) |
|  | Water | 1.26 | L | Ecoinvent v3.6 (UUID = c5adb1fb-872e-4446-a3bb-c4b61aa4bd45) | On-site measurement (water required for rice cooking) |
|  | Electricity | 0.82 | MJ | Ecoinvent v3.6 (UUID = 759b89bd-3aa6-42ad-b767-5bb9ef5d331d) | Electricity required for rice cooking, using q=mcΔT, temperature change 80˚C to boil water, simmer for 20 mins with a 250W heater, then E=Pt |
|  | Water | 0.84 | L | Ecoinvent v3.6 (UUID = c5adb1fb-872e-4446-a3bb-c4b61aa4bd45) | Water use for autoclave adapted from Garcia et al. 2012 [8] |
|  | Electricity | 4.83 | MJ | Ecoinvent v3.6 (UUID = 759b89bd-3aa6-42ad-b767-5bb9ef5d331d) | Energy use for autoclave adapted from Garcia et al. 2012 [8] |
|  | Polypropylene, granulate | 0.03 | kg | Ecoinvent v3.6 (UUID = 66ca2f38-5e51-4546-83c0-d7cef0c55c7c) | Modelling an autoclavable bag  On-site measurement of 8kg of substrate per bag |
|  | Latex | 0.002 | kg | Ecoinvent v3.6 (UUID = b0cd6c3d-d1b6-42f4-8e58-ca6d4d6c3e18) | Modelling nitrile gloves  On-site measurement of 1 pair |
|  | Water | 0.038 | L | Ecoinvent v3.6 (UUID = c5adb1fb-872e-4446-a3bb-c4b61aa4bd45) | On-site measurement (water to make 70% alcohol spray) |
|  | Ethanol | 0.088 | L | Ecoinvent v3.6 (UUID = 3a0d77a5-6488-4977-966a-731350be864a) | On-site measurement (ethanol to make 70% alcohol spray) |
|  | Electricity | 0.1 | MJ | Ecoinvent v3.6 (UUID = 759b89bd-3aa6-42ad-b767-5bb9ef5d331d) | Energy use of laminar flow hood, data from laminar flow hood technical data sheet [9] |
|  | Electricity | 2.04 | MJ | Ecoinvent v3.6 (UUID = 759b89bd-3aa6-42ad-b767-5bb9ef5d331d) | Energy use of lab incubator, data from laminar flow hood technical data sheet [10] |
| Mixing | Grain spawn | 2.52 | kg | Inoculated grain from grain spawn preparation | On-site measurement |
|  | Sterile substrate | 22.72 | kg | Sterilised substrate from substrate sterilisation | On-site measurement |
|  | Polyethylene, high density | 0.02 | kg | Ecoinvent v3.6 (UUID = b685192a-e033-421e-9074-8fe7cb176046) | Modelling plastic reusable mould, 1.2 square meters of HDPE with a thickness of 1 mm, used 50 times |
|  | Polyethylene, low density | 0.01 | kg | Ecoinvent v3.6 (UUID = 947a85fa-114e-4d9e-b36c-3293e48ea5ae) | Modelling parafilm sheet over the mould, 2 square meters |
|  | Ethanol | 0.07 | L | Ecoinvent v3.6 (UUID = 3a0d77a5-6488-4977-966a-731350be864a) | On-site measurement (ethanol to make 70% alcohol spray) |
|  | Water | 0.03 | L | Ecoinvent v3.6 (UUID = c5adb1fb-872e-4446-a3bb-c4b61aa4bd45) | On-site measurement (water to make 70% alcohol spray) |
|  | Latex | 0.002 | kg | Ecoinvent v3.6 (UUID = b0cd6c3d-d1b6-42f4-8e58-ca6d4d6c3e18) | Modelling nitrile gloves  On-site measurement of 1 pair |
|  | Electricity | 0.24 | MJ | Ecoinvent v3.6 (UUID = 759b89bd-3aa6-42ad-b767-5bb9ef5d331d) | Energy use of laminar flow hood, data from laminar flow hood technical data sheet [9] |
| Incubation | Mixed composite | 26.50 | kg | Mixed composite from mixing stage | On-site measurement |
|  | Electricity | 22.04 | MJ | Ecoinvent v3.6 (UUID = 759b89bd-3aa6-42ad-b767-5bb9ef5d331d) | Energy use of lab incubator, data from laminar flow hood technical data sheet [10] |
| Drying | Colonised composite | 26.50 | kg | Colonised composite following incubation | On-site measurement |
|  | Electricity | 25.9 | MJ | Ecoinvent v3.6 (UUID = 759b89bd-3aa6-42ad-b767-5bb9ef5d331d) | Energy use of laboratory oven, data from laboratory oven technical data sheet [11] |

*Table 2: Life Cycle Inventory: Life cycle inventory for the laboratory-scale production of 1 kg of mycelium-based composites (MBCs) using bark as a substrate for the scenario where the substrate is transported 10km. Inputs are categorised by process stages, with quantities, units, and data sources specified. Where possible, inputs specific to the GB market were selected for the life cycle inventory.*

Life Cycle Inventory Beech Wood Sawdust MBC:

| Process | Item | Quantity | Unit | Data source process | Data source quantity |
| --- | --- | --- | --- | --- | --- |
| Substrate preparation | Beech wood sawdust | 6.20 | Kg | Ecoinvent v3.6  (UUID = 7fe99768-d571-4bc2-a272-7df585bd0d48) | On-site measurement |
|  | Transport | 1.24 | Tonnes * kg | Ecoinvent v3.6  (UUID = f4e8b38b-d6c7-423b-bc9c-13b4a13d55f8) | Assume distance of 10km with a 16 tonne lorry filled with wood chips to the payload of 10 tonnes |
|  | Carbon | 8.02 | Kg | Ecoinvent v3.6  (UUID = cc6a1abb-b123-4ca6-8f16-38209df609be) | Biogenic carbon accounting follows the EN 16485:2023 guidelines[1]. The biogenic carbon stored in the OSB was calculated following the EN  16449:201448 standard [2], according to the formula: Pco2=4412×cf×m1+w100  , where Pco2 is the biogenic carbon storage in the sawdust (kg  CO2 eq), cf is the carbon fractional content of the sawdust [3,4], m is the mass of the sawdust, and w is the moisture  content of the sawdust. |
| Substrate soaking | Water | 49.99 | L | Ecoinvent v3.6 (UUID = c5adb1fb-872e-4446-a3bb-c4b61aa4bd45) | On-site measurement |
|  | Beech wood sawdust | 6.20 | kg | From substrate preparation | On-site measurement |
| Substrate sterilisation | Soaked sawdust | 24.99 | kg | From substrate soaking | On-site measurement (73% increase in mass of substrate upon soaking) |
|  | Electricity | 7.44 | MJ | Ecoinvent v3.6 (UUID = 759b89bd-3aa6-42ad-b767-5bb9ef5d331d) | Energy use for autoclave adapted from Garcia et al. 2012 [8] |
|  | Water | 8.27 | L | Ecoinvent v3.6 (UUID = c5adb1fb-872e-4446-a3bb-c4b61aa4bd45) | Water use for autoclave adapted from Garcia et al. 2012 [8] |
|  | Wheat flour | 2.78 | kg | Ecoinvent v3.6 (UUID = c7b1f264-abe2-47c4-8fcf-db3229228485) | On-site measurement |
|  | Polypropylene granulate | 0.10 | kg | Ecoinvent v3.6 (UUID = 66ca2f38-5e51-4546-83c0-d7cef0c55c7c) | Modelling an autoclavable bag  On-site measurement of 8kg of substrate per bag |
| Grain spawn preparation | Rice | 0.77 | kg | Ecoinvent v3.6 (UUID = 922db745-4df5-4b82-bbb6-98e81e4007d1) | On-site measurement (dry rice to cooked rice ratio 75:225) |
|  | Water | 1.54 | L | Ecoinvent v3.6 (UUID = c5adb1fb-872e-4446-a3bb-c4b61aa4bd45) | On-site measurement (water required for rice cooking) |
|  | Electricity | 1.00 | MJ | Ecoinvent v3.6 (UUID = 759b89bd-3aa6-42ad-b767-5bb9ef5d331d) | Electricity required for rice cooking, using q=mcΔT, temperature change 80˚C to boil water, simmer for 20 mins with a 250W heater, then E=Pt |
|  | Water | 0.93 | L | Ecoinvent v3.6 (UUID = c5adb1fb-872e-4446-a3bb-c4b61aa4bd45) | Water use for autoclave adapted from Garcia et al. 2012 [8] |
|  | Electricity | 5.38 | MJ | Ecoinvent v3.6 (UUID = 759b89bd-3aa6-42ad-b767-5bb9ef5d331d) | Energy use for autoclave adapted from Garcia et al. 2012 [8] |
|  | Polypropylene, granulate | 0.03 | kg | Ecoinvent v3.6 (UUID = 66ca2f38-5e51-4546-83c0-d7cef0c55c7c) | Modelling an autoclavable bag  On-site measurement of 8kg of substrate per bag |
|  | Latex | 0.002 | kg | Ecoinvent v3.6 (UUID = b0cd6c3d-d1b6-42f4-8e58-ca6d4d6c3e18) | Modelling nitrile gloves  On-site measurement of 1 pair |
|  | Water | 0.038 | L | Ecoinvent v3.6 (UUID = c5adb1fb-872e-4446-a3bb-c4b61aa4bd45) | On-site measurement (water to make 70% alcohol spray) |
|  | Ethanol | 0.088 | L | Ecoinvent v3.6 (UUID = 3a0d77a5-6488-4977-966a-731350be864a) | On-site measurement (ethanol to make 70% alcohol spray) |
|  | Electricity | 0.1 | MJ | Ecoinvent v3.6 (UUID = 759b89bd-3aa6-42ad-b767-5bb9ef5d331d) | Energy use of laminar flow hood, data from laminar flow hood technical data sheet [9] |
|  | Electricity | 2.27 | MJ | Ecoinvent v3.6 (UUID = 759b89bd-3aa6-42ad-b767-5bb9ef5d331d) | Energy use of lab incubator, data from laminar flow hood technical data sheet [10] |
| Mixing | Grain spawn | 3.09 | kg | Inoculated grain from grain spawn preparation | On-site measurement |
|  | Sterile substrate | 27.77 | kg | Sterilised substrate from substrate sterilisation | On-site measurement |
|  | Polyethylene, high density | 0.02 | kg | Ecoinvent v3.6 (UUID = b685192a-e033-421e-9074-8fe7cb176046) | Modelling plastic reusable mould, 1.2 square meters of HDPE with a thickness of 1 mm, used 50 times |
|  | Polyethylene, low density | 0.01 | kg | Ecoinvent v3.6 (UUID = 947a85fa-114e-4d9e-b36c-3293e48ea5ae) | Modelling parafilm sheet over the mould, 2 square meters |
|  | Ethanol | 0.07 | L | Ecoinvent v3.6 (UUID = 3a0d77a5-6488-4977-966a-731350be864a) | On-site measurement (ethanol to make 70% alcohol spray) |
|  | Water | 0.03 | L | Ecoinvent v3.6 (UUID = c5adb1fb-872e-4446-a3bb-c4b61aa4bd45) | On-site measurement (water to make 70% alcohol spray) |
|  | Latex | 0.002 | kg | Ecoinvent v3.6 (UUID = b0cd6c3d-d1b6-42f4-8e58-ca6d4d6c3e18) | Modelling nitrile gloves  On-site measurement of 1 pair |
|  | Electricity | 0.24 | MJ | Ecoinvent v3.6 (UUID = 759b89bd-3aa6-42ad-b767-5bb9ef5d331d) | Energy use of laminar flow hood, data from laminar flow hood technical data sheet [9] |
| Incubation | Mixed composite | 30.86 | kg | Mixed composite from mixing stage | On-site measurement |
|  | Electricity | 22.04 | MJ | Ecoinvent v3.6 (UUID = 759b89bd-3aa6-42ad-b767-5bb9ef5d331d) | Energy use of lab incubator, data from laminar flow hood technical data sheet [10] |
| Drying | Colonised composite | 30.86 | kg | Colonised composite following incubation | On-site measurement |
|  | Electricity | 25.9 | MJ | Ecoinvent v3.6 (UUID = 759b89bd-3aa6-42ad-b767-5bb9ef5d331d) | Energy use of laboratory oven, data from laboratory oven technical data sheet [11] |

*Table 3: Life Cycle Inventory: Life cycle inventory for the laboratory-scale production of 1 kg of mycelium-based composites (MBCs) using beech wood sawdust as a substrate for the scenario where the substrate is transported 10km. Inputs are categorised by process stages, with quantities, units, and data sources specified. Where possible, inputs specific to the GB market were selected for the life cycle inventory.*

Life Cycle Inventory Hemp-shiv MBC:

| Process | Item | Quantity | Unit | Data source process | Data source quantity |
| --- | --- | --- | --- | --- | --- |
| Substrate preparation | Hemp shiv | 5.69 | kg | Ecoinvent v3.6  (UUID = 7fe99768-d571-4bc2-a272-7df585bd0d48) | On-site measurement |
|  | Transport | 1.14 | Tonnes * kg | Ecoinvent v3.6  (UUID = f4e8b38b-d6c7-423b-bc9c-13b4a13d55f8) | Assume distance of 10km with a 16 tonne lorry filled with wood chips to the payload of 10 tonnes |
|  | Carbon | 8.55 | kg | Ecoinvent v3.6  (UUID = cc6a1abb-b123-4ca6-8f16-38209df609be) | Biogenic carbon accounting follows the EN 16485:2023 guidelines[1]. The biogenic carbon stored in the OSB was calculated following the EN  16449:201448 standard [2], according to the formula: Pco2=4412×cf×m1+w100  , where Pco2 is the biogenic carbon storage in the hemp (kg  CO2 eq), cf is the carbon fractional content of the hemp [3,6], m is the mass of the hemp, and w is the moisture  content of the hemp. |
| Substrate soaking | Water | 45.73 | L | Ecoinvent v3.6 (UUID = c5adb1fb-872e-4446-a3bb-c4b61aa4bd45) | On-site measurement |
|  | Hemp shiv | 5.69 | kg | From substrate preparation | On-site measurement |
| Substrate sterilisation | Soaked hemp shiv | 22.87 | kg | From substrate soaking | On-site measurement (73% increase in mass of substrate upon soaking) |
|  | Electricity | 6.96 | MJ | Ecoinvent v3.6 (UUID = 759b89bd-3aa6-42ad-b767-5bb9ef5d331d) | Energy use for autoclave adapted from Garcia et al. 2012 [8] |
|  | Water | 7.73 | L | Ecoinvent v3.6 (UUID = c5adb1fb-872e-4446-a3bb-c4b61aa4bd45) | Water use for autoclave adapted from Garcia et al. 2012 [8] |
|  | Wheat flour | 2.54 | kg | Ecoinvent v3.6 (UUID = c7b1f264-abe2-47c4-8fcf-db3229228485) | On-site measurement |
|  | Polypropylene granulate | 0.10 | kg | Ecoinvent v3.6 (UUID = 66ca2f38-5e51-4546-83c0-d7cef0c55c7c) | Modelling an autoclavable bag  On-site measurement of 8kg of substrate per bag |
| Grain spawn preparation | Rice | 0.71 | kg | Ecoinvent v3.6 (UUID = 922db745-4df5-4b82-bbb6-98e81e4007d1) | On-site measurement (dry rice to cooked rice ratio 75:225) |
|  | Water | 1.41 | L | Ecoinvent v3.6 (UUID = c5adb1fb-872e-4446-a3bb-c4b61aa4bd45) | On-site measurement (water required for rice cooking) |
|  | Electricity | 0.91 | MJ | Ecoinvent v3.6 (UUID = 759b89bd-3aa6-42ad-b767-5bb9ef5d331d) | Electricity required for rice cooking, using q=mcΔT, temperature change 80˚C to boil water, simmer for 20 mins with a 250W heater, then E=Pt |
|  | Water | 0.87 | L | Ecoinvent v3.6 (UUID = c5adb1fb-872e-4446-a3bb-c4b61aa4bd45) | Water use for autoclave adapted from Garcia et al. 2012 [8] |
|  | Electricity | 5.02 | MJ | Ecoinvent v3.6 (UUID = 759b89bd-3aa6-42ad-b767-5bb9ef5d331d) | Energy use for autoclave adapted from Garcia et al. 2012 [8] |
|  | Polypropylene, granulate | 0.03 | kg | Ecoinvent v3.6 (UUID = 66ca2f38-5e51-4546-83c0-d7cef0c55c7c) | Modelling an autoclavable bag  On-site measurement of 8kg of substrate per bag |
|  | Latex | 0.002 | kg | Ecoinvent v3.6 (UUID = b0cd6c3d-d1b6-42f4-8e58-ca6d4d6c3e18) | Modelling nitrile gloves  On-site measurement of 1 pair |
|  | Water | 0.038 | L | Ecoinvent v3.6 (UUID = c5adb1fb-872e-4446-a3bb-c4b61aa4bd45) | On-site measurement (water to make 70% alcohol spray) |
|  | Ethanol | 0.088 | L | Ecoinvent v3.6 (UUID = 3a0d77a5-6488-4977-966a-731350be864a) | On-site measurement (ethanol to make 70% alcohol spray) |
|  | Electricity | 0.1 | MJ | Ecoinvent v3.6 (UUID = 759b89bd-3aa6-42ad-b767-5bb9ef5d331d) | Energy use of laminar flow hood, data from laminar flow hood technical data sheet [9] |
|  | Electricity | 2.13 | MJ | Ecoinvent v3.6 (UUID = 759b89bd-3aa6-42ad-b767-5bb9ef5d331d) | Energy use of lab incubator, data from laminar flow hood technical data sheet [10] |
| Mixing | Grain spawn | 2.82 | kg | Inoculated grain from grain spawn preparation | On-site measurement |
|  | Sterile substrate | 25.41 | kg | Sterilised substrate from substrate sterilisation | On-site measurement |
|  | Polyethylene, high density | 0.02 | kg | Ecoinvent v3.6 (UUID = b685192a-e033-421e-9074-8fe7cb176046) | Modelling plastic reusable mould, 1.2 square meters of HDPE with a thickness of 1 mm, used 50 times |
|  | Polyethylene, low density | 0.01 | kg | Ecoinvent v3.6 (UUID = 947a85fa-114e-4d9e-b36c-3293e48ea5ae) | Modelling parafilm sheet over the mould, 2 square meters |
|  | Ethanol | 0.07 | L | Ecoinvent v3.6 (UUID = 3a0d77a5-6488-4977-966a-731350be864a) | On-site measurement (ethanol to make 70% alcohol spray) |
|  | Water | 0.03 | L | Ecoinvent v3.6 (UUID = c5adb1fb-872e-4446-a3bb-c4b61aa4bd45) | On-site measurement (water to make 70% alcohol spray) |
|  | Latex | 0.002 | kg | Ecoinvent v3.6 (UUID = b0cd6c3d-d1b6-42f4-8e58-ca6d4d6c3e18) | Modelling nitrile gloves  On-site measurement of 1 pair |
|  | Electricity | 0.24 | MJ | Ecoinvent v3.6 (UUID = 759b89bd-3aa6-42ad-b767-5bb9ef5d331d) | Energy use of laminar flow hood, data from laminar flow hood technical data sheet [9] |
| Incubation | Mixed composite | 28.23 | kg | Mixed composite from mixing stage | On-site measurement |
|  | Electricity | 22.04 | MJ | Ecoinvent v3.6 (UUID = 759b89bd-3aa6-42ad-b767-5bb9ef5d331d) | Energy use of lab incubator, data from laminar flow hood technical data sheet [10] |
| Drying | Colonised composite | 28.23 | kg | Colonised composite following incubation | On-site measurement |
|  | Electricity | 25.9 | MJ | Ecoinvent v3.6 (UUID = 759b89bd-3aa6-42ad-b767-5bb9ef5d331d) | Energy use of laboratory oven, data from laboratory oven technical data sheet [11] |

*Table 4: Life Cycle Inventory: Life cycle inventory for the laboratory-scale production of 1 kg of mycelium-based composites (MBCs) using hemp-shiv as a substrate for the scenario where the substrate is transported 10km. Inputs are categorised by process stages, with quantities, units, and data sources specified. Where possible, inputs specific to the GB market were selected for the life cycle inventory.*

Life Cycle Inventory Straw MBC:

| Process | Item | Quantity | Unit | Data source process | Data source quantity |
| --- | --- | --- | --- | --- | --- |
| Substrate preparation | Wheat straw | 1.40 | kg | Ecoinvent v3.6  (UUID = 7fe99768-d571-4bc2-a272-7df585bd0d48) | On-site measurement |
|  | Transport | 0.28 | Tonnes * kg | Ecoinvent v3.6  (UUID = f4e8b38b-d6c7-423b-bc9c-13b4a13d55f8) | Assume distance of 10km with a 16 tonne lorry filled with wood chips to the payload of 10 tonnes |
|  | Carbon | 1.81 | kg | Ecoinvent v3.6  (UUID = cc6a1abb-b123-4ca6-8f16-38209df609be) | Biogenic carbon accounting follows the EN 16485:2023 guidelines[1]. The biogenic carbon stored in the OSB was calculated following the EN  16449:201448 standard [2], according to the formula: Pco2=4412×cf×m1+w100  , where Pco2 is the biogenic carbon storage in the straw (kg  CO2 eq), cf is the carbon fractional content of the straw [3,7], m is the mass of the straw, and w is the moisture  content of the straw. |
| Substrate soaking | Water | 19.47 | L | Ecoinvent v3.6 (UUID = c5adb1fb-872e-4446-a3bb-c4b61aa4bd45) | On-site measurement |
|  | Wheat straw | 1.40 | kg | From substrate preparation | On-site measurement |
| Substrate sterilisation | Soaked wheat straw | 9.73 | kg | From substrate soaking | On-site measurement (73% increase in mass of substrate upon soaking) |
|  | Electricity | 2.88 | MJ | Ecoinvent v3.6 (UUID = 759b89bd-3aa6-42ad-b767-5bb9ef5d331d) | Energy use for autoclave adapted from Garcia et al. 2012 [8] |
|  | Water | 3.20 | L | Ecoinvent v3.6 (UUID = c5adb1fb-872e-4446-a3bb-c4b61aa4bd45) | Water use for autoclave adapted from Garcia et al. 2012 [8] |
|  | Wheat flour | 1.08 | kg | Ecoinvent v3.6 (UUID = c7b1f264-abe2-47c4-8fcf-db3229228485) | On-site measurement |
|  | Polypropylene granulate | 0.04 | kg | Ecoinvent v3.6 (UUID = 66ca2f38-5e51-4546-83c0-d7cef0c55c7c) | Modelling an autoclavable bag  On-site measurement of 8kg of substrate per bag |
| Grain spawn preparation | Rice | 0.30 | kg | Ecoinvent v3.6 (UUID = 922db745-4df5-4b82-bbb6-98e81e4007d1) | On-site measurement (dry rice to cooked rice ratio 75:225) |
|  | Water | 0.60 | L | Ecoinvent v3.6 (UUID = c5adb1fb-872e-4446-a3bb-c4b61aa4bd45) | On-site measurement (water required for rice cooking) |
|  | Electricity | 0.39 | MJ | Ecoinvent v3.6 (UUID = 759b89bd-3aa6-42ad-b767-5bb9ef5d331d) | Electricity required for rice cooking, using q=mcΔT, temperature change 80˚C to boil water, simmer for 20 mins with a 250W heater, then E=Pt |
|  | Water | 0.36 | L | Ecoinvent v3.6 (UUID = c5adb1fb-872e-4446-a3bb-c4b61aa4bd45) | Water use for autoclave adapted from Garcia et al. 2012 [8] |
|  | Electricity | 2.08 | MJ | Ecoinvent v3.6 (UUID = 759b89bd-3aa6-42ad-b767-5bb9ef5d331d) | Energy use for autoclave adapted from Garcia et al. 2012 [8] |
|  | Polypropylene, granulate | 0.01 | kg | Ecoinvent v3.6 (UUID = 66ca2f38-5e51-4546-83c0-d7cef0c55c7c) | Modelling an autoclavable bag  On-site measurement of 8kg of substrate per bag |
|  | Latex | 0.002 | kg | Ecoinvent v3.6 (UUID = b0cd6c3d-d1b6-42f4-8e58-ca6d4d6c3e18) | Modelling nitrile gloves  On-site measurement of 1 pair |
|  | Water | 0.038 | L | Ecoinvent v3.6 (UUID = c5adb1fb-872e-4446-a3bb-c4b61aa4bd45) | On-site measurement (water to make 70% alcohol spray) |
|  | Ethanol | 0.088 | L | Ecoinvent v3.6 (UUID = 3a0d77a5-6488-4977-966a-731350be864a) | On-site measurement (ethanol to make 70% alcohol spray) |
|  | Electricity | 0.1 | MJ | Ecoinvent v3.6 (UUID = 759b89bd-3aa6-42ad-b767-5bb9ef5d331d) | Energy use of laminar flow hood, data from laminar flow hood technical data sheet [9] |
|  | Electricity | 0.88 | MJ | Ecoinvent v3.6 (UUID = 759b89bd-3aa6-42ad-b767-5bb9ef5d331d) | Energy use of lab incubator, data from laminar flow hood technical data sheet [10] |
| Mixing | Grain spawn | 1.20 | kg | Inoculated grain from grain spawn preparation | On-site measurement |
|  | Sterile substrate | 10.82 | kg | Sterilised substrate from substrate sterilisation | On-site measurement |
|  | Polyethylene, high density | 0.02 | kg | Ecoinvent v3.6 (UUID = b685192a-e033-421e-9074-8fe7cb176046) | Modelling plastic reusable mould, 1.2 square meters of HDPE with a thickness of 1 mm, used 50 times |
|  | Polyethylene, low density | 0.01 | kg | Ecoinvent v3.6 (UUID = 947a85fa-114e-4d9e-b36c-3293e48ea5ae) | Modelling parafilm sheet over the mould, 2 square meters |
|  | Ethanol | 0.07 | L | Ecoinvent v3.6 (UUID = 3a0d77a5-6488-4977-966a-731350be864a) | On-site measurement (ethanol to make 70% alcohol spray) |
|  | Water | 0.03 | L | Ecoinvent v3.6 (UUID = c5adb1fb-872e-4446-a3bb-c4b61aa4bd45) | On-site measurement (water to make 70% alcohol spray) |
|  | Latex | 0.002 | kg | Ecoinvent v3.6 (UUID = b0cd6c3d-d1b6-42f4-8e58-ca6d4d6c3e18) | Modelling nitrile gloves  On-site measurement of 1 pair |
|  | Electricity | 0.24 | MJ | Ecoinvent v3.6 (UUID = 759b89bd-3aa6-42ad-b767-5bb9ef5d331d) | Energy use of laminar flow hood, data from laminar flow hood technical data sheet [9] |
| Incubation | Mixed composite | 12.02 | kg | Mixed composite from mixing stage | On-site measurement |
|  | Electricity | 22.04 | MJ | Ecoinvent v3.6 (UUID = 759b89bd-3aa6-42ad-b767-5bb9ef5d331d) | Energy use of lab incubator, data from laminar flow hood technical data sheet [10] |
| Drying | Colonised composite | 26.50 | kg | Colonised composite following incubation | On-site measurement |
|  | Electricity | 25.9 | MJ | Ecoinvent v3.6 (UUID = 759b89bd-3aa6-42ad-b767-5bb9ef5d331d) | Energy use of laboratory oven, data from laboratory oven technical data sheet [11] |

*Table 5: Life Cycle Inventory: Life cycle inventory for the laboratory-scale production of 1 kg of mycelium-based composites (MBCs) using straw as a substrate for the scenario where the substrate is transported 10km. Inputs are categorised by process stages, with quantities, units, and data sources specified. Where possible, inputs specific to the GB market were selected for the life cycle inventory.*

| Transport method | Data source |
| --- | --- |
| Rail (freight train) | Ecoinvent v3.6 UUID = 0ace02fa-eca5-482d-a829-c18e46a52db4 |
| Ship (freight, sea, container ship) | Ecoinvent v3.6 UUID = 2741cea8-327f-4e0f-9401-b10858dc68f8 |

*Table 6: Data source items implemented in OpenLCA using the Ecoinvent v3.6 database for additional transport scenarios.*

References:

[1] EN16485:2023 Round and sawn timber- Environmental Product Declarations- Product category rules for wood and wood-based products for use in construction (2023)

[2] EN16449:2014 Wood and wood-based products - Calculation of the biogenic carbon content of wood and conversion to carbon dioxide (2014)

[3] Eggleston, HS and Buendia, Leandro and Miwa, Kyoko and Ngara, Todd and Tanabe, Kiyoto. 2006 IPCC guidelines for national greenhouse gas inventories. (2006)

[4] Lamlom, S. H., & Savidge, R. A. (2003). A reassessment of carbon content in wood: variation within and between 41 North American species. *Biomass and Bioenergy*, *25*(4), 381-388.

[5] Sobol, Łukasz, Dominika Sabat, and Arkadiusz Dyjakon. "Assessment of bark properties from various tree species in terms of its hydrophobicity and energy suitability." *Energies* 16.18 (2023): 6586.

[6] De Beus, N., M. Stratmann, and M. Carus. "Carbon Storage in Hemp and Wood Raw Materials for Construction Materials." *Nova Institute for Ecology and Innovation* (2023).

[7] Kumar, Manoj & Singh, R. P. & Panigrahy, S. & Raghubanshi, Akhilesh. (2014). Carbon density and accumulation in agroecosystem of Indo-Gangetic Plains and Vindhyan highlands, India. Environmental monitoring and assessment. 186. 10.1007/ s10661-014-3752-3. Table 3

[8] García, A. et al. Biological treatment of the organic fibre from the autoclaving of municipal solid wastes; preliminary results. Biosyst. Eng. 112, 335343, DOI: 10.1016/j.biosystemseng.2012.05.005 (2012)

[9] Ossila BV. Laminar Flow Hood Technical Manual, Version 1.0.A. Leiden, The Netherlands (2023)

[10] POL-EKO-APARATURA. Laboratory Incubator CLN 115 Smart: Technical Data Sheet. Poland (2023)

[11] Weiss Technik GmbH. Industrial Laboratory Ovens: Technical Data Sheet. Reiskirchen, Germany (2023)
